# Genome Mining for Antimicrobial Compounds in Wild Marine Animals-Associated Enterococci

**DOI:** 10.3390/md19060328

**Published:** 2021-06-06

**Authors:** Janira Prichula, Muriel Primon-Barros, Romeu C. Z. Luz, Ícaro M. S. Castro, Thiago G. S. Paim, Maurício Tavares, Rodrigo Ligabue-Braun, Pedro A. d’Azevedo, Jeverson Frazzon, Ana P. G. Frazzon, Adriana Seixas, Michael S. Gilmore

**Affiliations:** 1 Gram-Positive Cocci Laboratory, Federal University of Health Sciences of Porto Alegre (UFCSPA), Porto Alegre 90050-170, RS, Brazil; janirap@ufcspa.edu.br (J.P.); murielp@ufcspa.edu.br (M.P.-B.); romeulu@ufcspa.edu.br (R.C.Z.L.); icaromsc@ufcspa.edu.br (Í.M.S.C.); thiagopucrs@gmail.com (T.G.S.P.); pedroaze@ufcspa.edu.br (P.A.d.); 2 Centro de Estudos Costeiros, Limnológicos e Marinhos (CECLIMAR), Universidade Federal do Rio Grande do Sul (UFRGS), Campus Litoral Norte, Imbé 95625-000, RS, Brazil; mauricio.tavares@ufrgs.br; 3 Department of Pharmacosciences, UFCSPA, Porto Alegre 90050-170, RS, Brazil; rodrigolb@ufcspa.edu.br (R.L.-B.); adrianaseixas@ufcspa.edu.br (A.S.); 4 Food Science Institute, UFRGS, Porto Alegre 90035-003, RS, Brazil; jeverson.frazzon@ufrgs.br; 5 Department of Microbiology, Immunology and Parasitology, UFRGS, Porto Alegre 90050-170, RS, Brazil; ana.frazzon@ufrgs.br; 6 Massachusetts Eye and Ear Infirmary, Department of Ophthalmology, Harvard Medical School, Boston, MA 02114, USA; 7 Department of Microbiology, Harvard Medical School, Boston, MA 02115, USA

**Keywords:** enterococci, genome-wide analysis, bacteriocins, probiotics, wild marine species

## Abstract

New ecosystems are being actively mined for new bioactive compounds. Because of the large amount of unexplored biodiversity, bacteria from marine environments are especially promising. Further, host-associated microbes are of special interest because of their low toxicity and compatibility with host health. Here, we identified and characterized biosynthetic gene clusters encoding antimicrobial compounds in host-associated enterococci recovered from fecal samples of wild marine animals remote from human-affected ecosystems. Putative biosynthetic gene clusters in the genomes of 22 *Enterococcus* strains of marine origin were predicted using antiSMASH5 and Bagel4 bioinformatic software. At least one gene cluster encoding a putative bioactive compound precursor was identified in each genome. Collectively, 73 putative antimicrobial compounds were identified, including 61 bacteriocins (83.56%), 10 terpenes (13.70%), and 2 (2.74%) related to putative nonribosomal peptides (NRPs). Two of the species studied, *Enterococcus avium* and *Enterococcus mundtti*, are rare causes of human disease and were found to lack any known pathogenic determinants but yet possessed bacteriocin biosynthetic genes, suggesting possible additional utility as probiotics. Wild marine animal-associated enterococci from human-remote ecosystems provide a potentially rich source for new antimicrobial compounds of therapeutic and industrial value and potential probiotic application.

## 1. Introduction

Drug-resistant bacteria kill an estimated 700,000 people worldwide each year, and the discovery of new antimicrobial drugs is urgently needed [1,2,3]. This is motivating the search for new ecologies for novel natural products of potential therapeutic value. Human-proximal terrestrial life has been screened for diverse natural products to a much greater extent than larger but less accessible marine ecosystems. Blue biotechnology (or marine biotechnology) is an emerging field that investigates the rich diversity of bioactive molecules produced by marine organisms with potential industrial and therapeutic applications [4,5,6,7,8,9]. Early successes include compounds derived from a gastropod (e.g., ziconotide, commercial name Prialt [10]), sponge (e.g., eribulin mesylate, commercial name Halaven [11]), cyanobacteria (e.g., dolastatin 10 [12], apratoxin A [13], and barbamide [14]), fungi (e.g., penicillipyrone A and B [15], and aszonapyrone A [16]), algae (e.g., neolaurene [17] and diphlorethohydroxycarmalol (DPHC) [18]), and bacteria (e.g., salinosporamide A [19], abyssomicin C [20], forazoline A [21], and farnesylquinone [22].

Recently, host-associated microbes also have drawn attention as a potential source for low toxicity agents compatible with host health but active against pathogenic microbes [23,24]. It was, therefore, of interest to us to explore marine animals from remote habitats for host-associated microbes that encode novel natural product biosynthetic pathways. Further, we focused on host-associated enterococci, a genus of gut microbes associated with all classes of land animals studied [25], and with animals that have returned to the marine environment [26]. Although most enterococci exist as harmless commensals, some lineages of the species *Enterococcus faecalis* and *Enterococcus faecium* have emerged as leading causes of multidrug-resistant hospital infection [25,27,28,29,30].

Enterococci are known to produce bacteriocins with narrow to broad antimicrobial activity [31,32,33]. Bacteriocins have found use as natural antimicrobial agents so far, mainly in the food industry but could complement traditional antibiotics in controlling important human and animal pathogens [34,35]. Different classification schemes have been proposed for bacteriocins produced by Lactic Acid Bacteria (LAB), although still a subject of debate [33,36,37]. Class I bacteriocins are posttranslationally modified peptides with less than 10 kDa that require enzymatic modification during biosynthesis, and thereby, the molecules have uncommon amino acids and structures that impact their properties [36]. Class II bacteriocins are also less than 10 kDa, although they are heat stable and unmodified peptides [36] with the exception of disulfide bridging, circularization, and methionine formylation [33]. This class has been subclassified: IIa—pediocin-like bacteriocins; IIb—two-peptide bacteriocins; circular bacteriocins; leaderless; and other bacteriocins that do not fall into any of the recognized subclasses [33]. On the other hand, Class III bacteriocins are large-molecular-weight (more than 10 kDa) and heat-labile antimicrobial proteins usually composed of different domains [36]. Divergently, some authors have been classified circular bacteriocins as class IV [38] or as Class Ib [36] since these head-to-tail cyclized peptides whose N- and C-termini are linked by a peptide bond, thereby rendering a circular molecule [36].

The bacteriocins synthetized by enterococci, enterocins, are generally small molecular weight (20–60 amino acids), often post-translationally modified peptides with cationic, hydrophobic, and heat-stable properties [32,33,36]. They vary in their mode of action, activity spectrum (restricted or broad), molecular mass, biochemical properties, and genetic origin [33,39,40]. Most known enterocins are produced by *E. faecium* and *E. faecalis*, but a few peptides have also been isolated from *Enterococcus mundtii*, *Enterococcus avium*, *Enterococcus durans*, *Enterococcus hirae*, and *Enterococcus lactis* [33,38]. Most characterized enterocins derive from enterococci associated with food, waste, feces, and gastrointestinal tract of humans and other animals [32,33,41]. Few have been described from enterococci from wild ecologies [8,26,42,43,44].

Traditionally, new bioactive compounds have been identified by screening microorganism extracts for biological activity or by amplification of new genes using polymerase chain reaction (PCR) [45,46,47,48]. These screening strategies are limited by time-consuming and laborious test methods [24,49]. Advances in molecular biology, bioinformatics, and genomics have been providing important new tools for exploration and development [50,51,52]. Genome screening has identified a large pool of potential compounds encoded by biosynthetic gene clusters (BGCs) in DNA databases [1,53,54,55,56]. The identification of new BGCs may be performed by applying algorithms based on indicators (e.g., evolutionary hallmarks, signature protein domains, and distant paralogs of primary metabolic enzymes) and using bioinformatic tools, such as antiSMASH5 [57] and BAGEL4 [58]. High throughput computational technologies are being used for screening, presumptive chemical elucidation, and understanding of activities and biological aspects of new compounds [7,24].

Therefore, genome mining may represent a fertile strategy for identifying new biomolecules for future therapeutic and industrial applications. In this sense, the aim of the present study was to examine 22 genomes of *Enterococcus* species isolated from fecal samples of 17 wild marine animals from remote ecologies for potential antimicrobial compounds and/or probiotics strains.

## 2. Results

### 2.1. Diversity of Wild Marine Animals Associated-Enterococci

The genomes of 22 *Enterococcus* spp. isolated from wild sea turtles, seabirds, and marine mammals were sequenced (Table 1). A summary of the sequencing statistics is presented in Supplementary Table S1. The genomes sizes were between 2.6–4.5 Mb, with GC contents ranging from 36.3% to 42.4%. All genomes share average nucleotide identities (ANI) above 95% with known species [59], confirming designation as *Enterococcus avium* (4.54%); *Enterococcus casseliflavus* (13.64%); *Enterococcus faecalis* (45.45%); *Enterococcus hirae* (27.27%); *Enterococcus lactis* (4.54%); *Enterococcus mundtii* (4.54%) species (Table 1; Supplementary Table S2).

### 2.2. Marine Enterococcal Genomes Harbor Diverse Biosynthetic Gene Clusters (BGCs) Coding for Antimicrobial Compounds

Two informatic packages, antiSMASH5 [57] and Bagel4 [58], accurately predict all known enterococcal bacteriocins whose properties have been well studied [32,33], including bacteriocin 31, bacteriocin T8, durancin Q, enterocin 96, enterocin1071A and 1071B, enterocin_A, enterocin B, enterocin CRL35, enterocin EJ97, enterocin SE-K4, enterocin P, enterocin Xα and Xβ, enterolysin A, hiracin JM79, mundticin KS, and others. This also includes the *E. faecalis* cytolysin, a highly divergent two-component lantipeptide-type bacteriocin active against nearly all Gram positives [60], which also possesses lytic activity for some eukaryotic cells [61]. Therefore, antiSMASH5 [57] and Bagel4 [58] were used to mine the genomes of all 22 genomes for putative bacteriocin biosynthesis operons (Supplementary Table S3). This analysis identified one or more gene clusters encoding a bioactive compound precursor in each genome. In total, 73 antimicrobial compound BGCs were predicted, including 61 (83.56%) bacteriocins, 10 (13.70%) related to terpene synthesis, and 2 (2.74%) related to putative nonribosomal peptides (NRPs). The NRPs biosynthetic gene clusters were found only in *E. lactis* genome (MP10-1), whereas terpene BGCs were found among *E. casseliflavus* (HT1-1, J2, J4), *E. hirae* (C7, DMW1-1, MP1-1, MP1-2, MP1-4, MP1-5), and *E. mundtii* (MP7-18) species (Supplementary Table S3). NRP and terpene BGCs were predicted only by antiSMASH5 [57], whereas bacteriocins were identified by both tools.

### 2.3. Diversity of Bacteriocins Genes among Wild Marine Animals-Associated Enterococci

**Figure 1:**
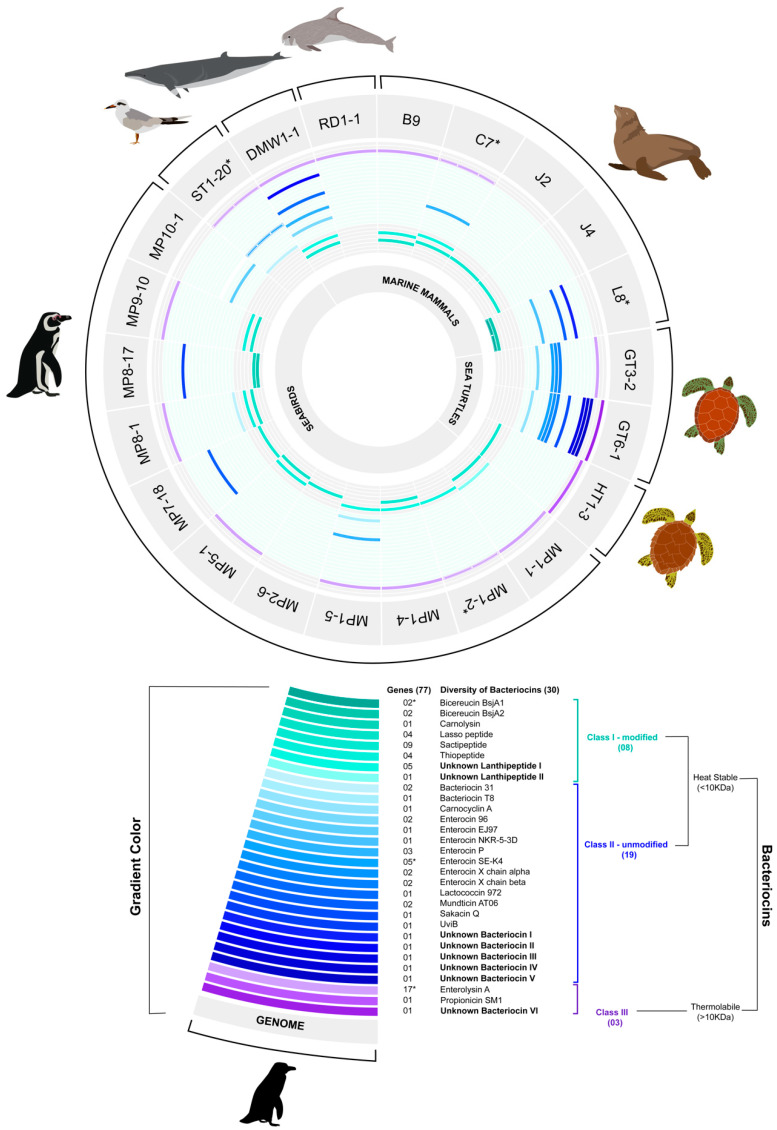
Biosynthetic bacteriocins genes were found within 22 *Enterococcus* spp. genomes from wild marine animals. The *Enterococcus* genomes are represented in the external circle (grey). Diversity of bacteriocin genes within 22 *Enterococcus* spp. genomes are represented by color gradients: Class I (green gradient) and Class II (blue gradient), and Class III (purple). * Genomes showing duplicated bacteriocin genes (rectangles indicate the number of these genes). The illustration was designed using a D3 and Adobe Illustrator.

A total of 30 unique bacteriocin species were identified, including 8 belonging to class I, 19 to class II, and 3 to class III (Figure 1). Although class II bacteriocins showed the greatest diversity, class III bacteriocins were most common and widely distributed. Interestingly, eight new putative bacteriocins with no significant identity to known peptides were found amongst marine enterococci genomes, including two new putative lanthipeptides (I and II) identified as class I, five unknown bacteriocins (I, II, III, IV, and V) identified as class II, and one unknown class III bacteriocin (VI) (Figure 1; Supplementary Table S4).

The most frequent class I bacteriocins were putative sactipeptides (*n* = 9), followed by unknown lanthipeptide 1 (*n* = 5), lasso peptides (*n* = 4), and thiopeptides (*n* = 4). Enterocin SE-K4 (*n* = 5) and enterocin P (*n* = 3) were the most frequent class II bacteriocins. In turn, the class III bacteriocin enterolysin A (*n* = 17) was the most frequent bacteriocin found in the 22 sequenced genomes (Figure 1).

Eight enterococcal genomes belonging to *E. hirae* (C7, DMW1-1, MP1-5), *E. avium* (L8), and *E. faecalis* (GT3-2, GT6-1, MP8-1, and ST1-20) species showed four or more bacteriocin biosynthetic genes (Figure 1). Four of these genomes (C7, DMW1-1, MP1-5, and MP8-1) encode bacteriocins belonging to three different classes (I, II, and III). Moreover, four enterococci genomes (C7, L8, ST1-20, and MP1-2) exhibited evidence of bacteriocin gene duplication (Figure 1; Supplementary Table S3). Because of their potentially new bacteriocins and/or amenability large-scale synthesis, putative class II and III bacteriocins were of special interest for further analysis.

### 2.4. Phylogenetic Relationship among Class II and III Bacteriocins Predicted from Wild Marine Animal-Associated Enterococcal Genomes

**Figure 2:**
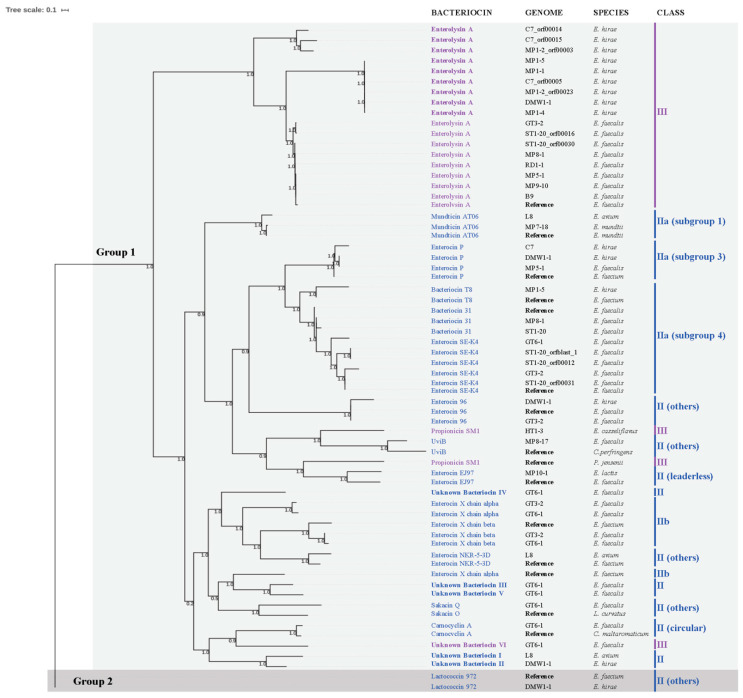
The phylogenetic relationships among bacteriocins (Class II and III) predicted for wild marine animals-associated enterococci genomes. The different groups are represented by grey colors (light grey: Group 1 and dark grey: Group 2). Class II is represented in blue and class III in purple (bold purple are enterolysins A from *E. hirae*, and regular purple are enterolysins A from *E. faecalis*). Unknown bacteriocins are highlighted in bold blue (I, II, III, IV, and V) and bold purple (VI).

To gain insights into the phylogeny of the 30 class II and 19 class III bacteriocins genes identified, phylogenetic analysis was performed (Figure 2) to determine their relationship (Supplementary Table S5) to 16 reference sequences in Bagel4 and Uniprot databases (Supplementary Table S6). This identified two groups with significant branch support (Figure 2). Group 1 included bacteriocins of both classes II and III. Class II bacteriocin gene clusters in Group 1 could be divided into subclasses a, b, and others. Included within each are: *IIa*) mundticin AT06, enterocin P, bacteriocin T8, bacteriocin 31, and enterocin SE-K4; *IIb*) enterocin X chain alpha, enterocin X chain beta; *II leaderless*) enterocin EJ97; *II circular bacteriocin*) carnocyclin A; *II other subclasses*) sakacin Q, enterocin 96, uviB, and enterocin NKR-5-3D; and unknown bacteriocins I, II, III, IV, and V. Class III bacteriocins in Group 1 included: enterolysin A, propionicin SM1, and unknown bacteriocin VI. In contrast, phylogenetic Group 2 included only the class II bacteriocin, lactococcin 972.

Interestingly, the 17 Class III enterolysin A-related sequences occurring in Group 1 could be grouped into three subclades. The first and second branches included sequences derived from *E. hirae* strains C7, MP1-1, MP1-2, MP1-4, MP1-5, DMW1-1, while the third branch included enterolysins A from *E. faecalis* strains GT3-2, ST1-20, MP8-1, RD1-1, MP5-1, MP9-10, and B9. The alignment of enterolysin A sequences within each branch (Supplementary Figures S1–S3) shows high similarity among them, although they have few conserved amino acids compared to the enterolysin A reference sequences (Supplementary Figure S4).

The alignment of the other bacteriocin sequences with reference sequences was performed (Supplementary Figures S5–S10). Among identities found were conserved motifs such as YGN and cysteine residues (all class IIa bacteriocins can be found in Supplementary Figure S6), and GxxxG or AxxxA motifs among class IIb and circular bacteriocin members (Supplementary Figures S7 and S8).

**Figure 3:**
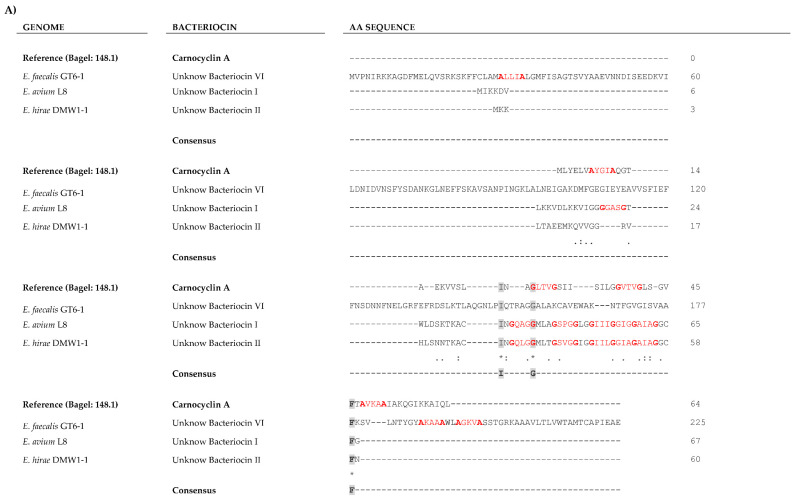

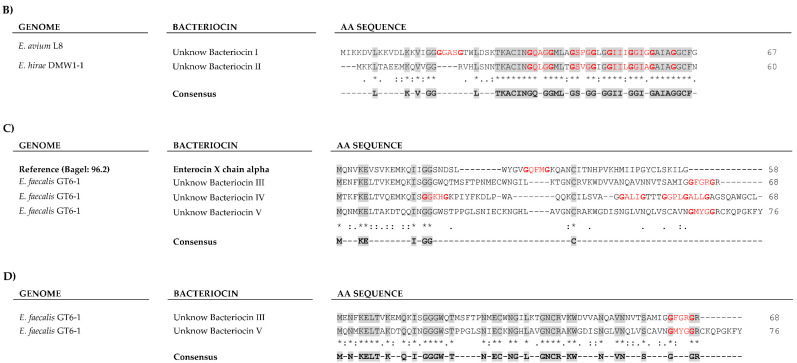
The alignment of putative unknown Class II bacteriocins and reference sequences using Clustal Omega software. (**A**) Alignment among I, II, VI, and carnocyclin A (reference) (Identity (*): 1.3%; Strongly similar (:): 2.2%; Weakly similar (.): 4.4%]. (**B**) The alignment between I and II [Identity (*): 55.22%; Strongly similar (:): 11.94%; Weakly similar (.) 10.45%]. (**C**) Alignment among III, VI, V, and enterocin Xα (reference) [Identity (*): 9.2%; Strongly similar (:): 11.8%; Weakly similar (.): 9.2%]. (**D**) Alignment between I and II [Identity (*): 43.4%; Strongly similar (:): 14.5%; Weakly similar (.) 11.8%). Identical residues are shaded in grey, and GxxxG or AxxxA motives are represented in red color. (-) Gaps introduced to optimize alignments. (*) Positions with a single conserved residue. (:) Conservation between groups with strongly similar properties. (.) Conservation between groups with weakly similar properties.

**Figure 4:**
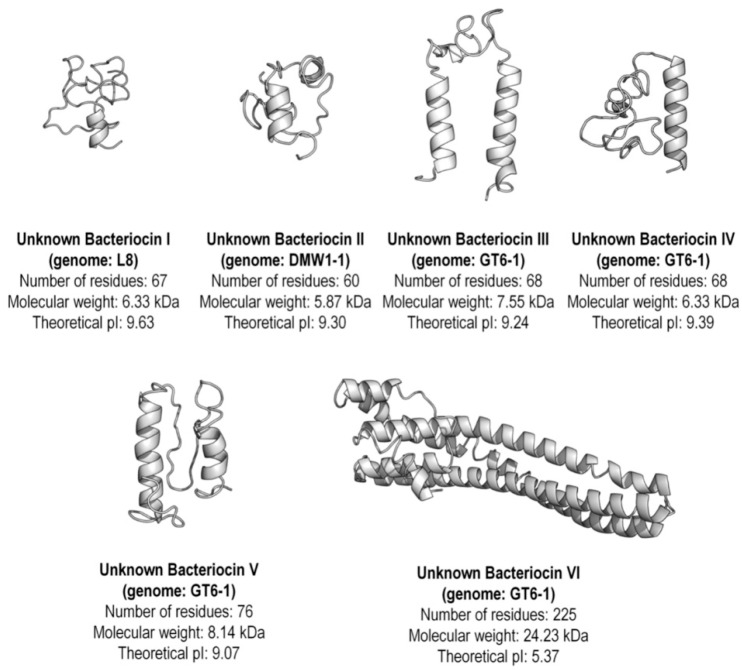
The structural modeling of unknown Class II enterococcal bacteriocins from wild marine animals.

New putative bacteriocins I, II, and VI showed greater similarity to carnocyclin A, while the unknown bacteriocins III, IV and V were more closely related to enterocin X chain alpha (Xα) (Figure 2). Alignment of unknown bacteriocins with carnocyclin A and Enterocin Xα reference sequences allowed detection of conserved amino acid residues and motifs such as GxxxG or AxxxA (Figure 3). Putative novel bacteriocins I, II, VI and carnocyclin A showed only 1.3% overall amino acid sequence identity (Figure 3A), whereas bacteriocins I and II share 55.22% identity between them (Figure 3B). Putative bacteriocins III, IV, and V, which were closely related to enterocin Xα, have 9.2% overall amino acid sequence identity (Figure 3C); and bacteriocins III and V share 43.4% identity between them (Figure 3D). Structural modeling of these putative class II and III bacteriocins using the I-TASSER [62] package to build models using a combination of fragment and ab initio model building [63] is shown in Figure 4. Insights into structural features are important for the biosynthesis, mode of action, and biological activity of bacteriocins. The molecular models are in agreement with the expected protein folds (mostly alpha-helices with coil regions). Likewise, the most divergent model (Bacteriocin VI) is also isolated in its group in the phylogenetic reconstruction, supporting its uniqueness among other unknown bacteriocins.

### 2.5. Detection of Genes Associated with Enhanced Enterococcal Virulence

**Figure 5:**
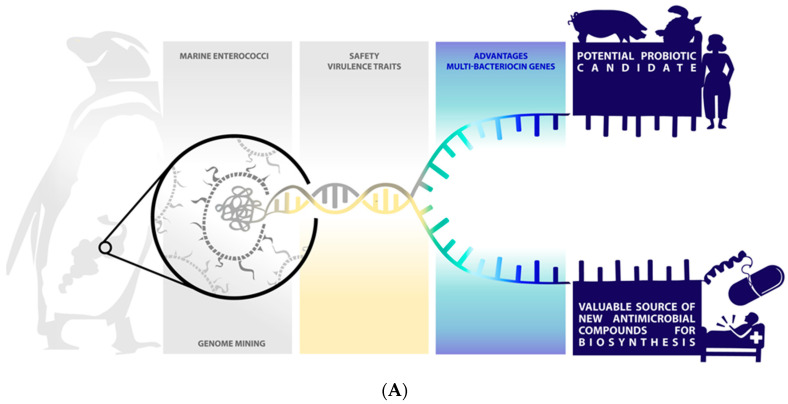

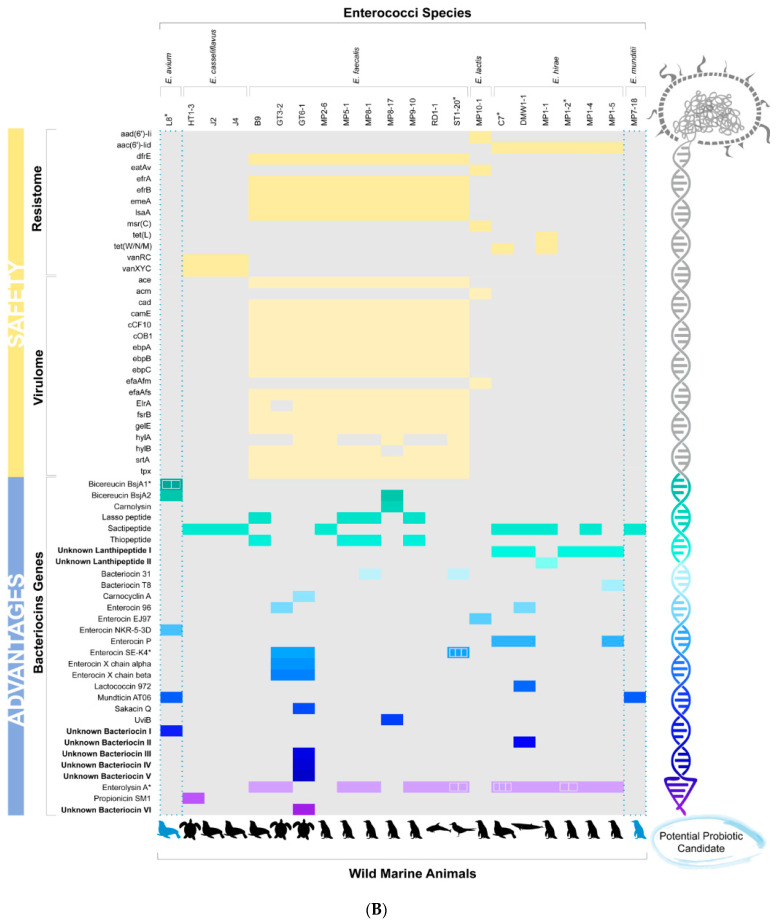
Wild marine animals-associated enterococci might represent a potentially valuable source of new compounds for biotechnological application and generation of new drug leads and potential probiotic application. (**A**) Scheme showing the main marine enterococci biotechnological applications suggested in this study. (**B**) Virulence markers analysis revealed potential probiotic enterococci from wild marine animals. Determinants of resistance (light yellow) and virulence (dark yellow) were associated with the results of in silico screening by bacteriocins (green, blue, and purple colors). * Genomes showing duplicated bacteriocin genes (rectangles are representing the number of these genes). Blue dash representing the potential probiotic candidate strains (L8 and MP7-18). The illustration was designed using D3, R software, and Adobe Illustrator.

Among the 22 genomes evaluated, *E. avium* (L8) and *E. mundtii* (MP7-18) were found to be devoid of determinants that have mainly been identified in *E. faecalis* and *E. faecium* strains associated with enhanced virulence (Figure 5A,B). All other enterococci strains possessed at least one potential virulence-associated trait (Figure 5B). As expected, these were most common in *E. faecalis*, where they have been most thoroughly studied. Some of these traits are encoded within the core genomes [25,26]. The unique *E. lactis* harbored *efa*Afm and *acm* genes, while all *E. faecalis* contained several genes associated with adhesion (*ace*, *efa*Afs), biofilm production (*ebp*A, *ebp*B and *ebp*C), proteases (*gel*E and *srt*A), protection against oxidative stress (*tpx*), and quorum sensing and sex pheromone (*cad*, *cam*E, *c*CF10, *c*OB1, and *fsr*B). *Enterococcus faecalis* genomes varied in the presence of hyaluronidase genes (*hyl*A and *hyl*B) and adhesion-associated gene (*Elr*A).

Resistome analysis (Figure 5B) revealed that all *E. casseliflavus* genomes (*n* = 3) possessed genes related to low-level vancomycin resistance (*van*RC and *van*XYC), as expected since these are part of the core genome for that species [64]. All *E. faecalis* genomes (*n* = 10) contained genes within the core genomes [26] conferring resistance to trimethoprim (*dfr*E); to macrolide, fluoroquinolone, and rifamycin (*efr*A and *efr*B); to pleuromutilin, lincosamide, and streptogramin (*lsa*A); and have a multidrug and toxic compound extrusion transporter (*eme*A). On the other hand, the unique *E. lactis* genome possessed genes related to the resistance to aminoglycosides (*aac*(6′)-Ii); to macrolide, lincosamide, streptogramin, tetracycline, oxazolidinone, phenicol, pleuromutilin (*eat*Av); and to macrolide, lincosamide, streptogramin (*msr*C). In addition, *E*. *hirae* genomes harbored genes related to aminoglycoside (*acc*(6′)-Iid; *n* = 6), and tetracycline [*tet*(W/N/M), *n* = 2; *tet*(L); *n* = 1] resistance.

## 3. Discussion

Microbes associated with marine animals from remote ecologies may be important sources for new tools to manage human and/or microbial interactions. In this study, we explored *Enterococcus* strains from the microbiota of wild sea birds, sea turtles, and marine mammals that range from the Antarctic to the coast of Brazil to identify potentially novel BGCs. These prospective BCGs were found in generalist species *E. faecalis*, as well as less common and less studied species, including *E. avium*, *E. casseliflavus*, *E. hirae*, *E. lactis*, and *E. mundtii*.

Putative bacteriocin genes were present in all enterococcal strains investigated, highlighting the competitive nature of the gut niche. Bacteriocin-encoding genes are known to be widely disseminated among enterococci species of different origins [33,54,55]. However, likely because of the novel environmental source of these strains, we found considerable diversity and novelty (Figure 1), with eight genomes possessing four or more bacteriocin gene clusters. This may be driven by variation in wild marine animal diets along migratory routes, combined with selection pressure for factors to control population structure and niche control in the host gut.

Enterococcal bacteriocins are of interest because of their antimicrobial activities, with activity against different Gram-positive and Gram-negative bacteria, including species of *Listeria*, *Clostridium*, *Staphylococcus*, *Streptococcus*, *Cutibacterium*, *Pseudomonas*, and *Salmonella* [6,33,34,65]. Enterocins have also been described as effective agents against antibiotic-resistant bacteria such as vancomycin-resistant enterococci (VRE) and methicillin-resistant *Staphylococcus aureus* (MRSA) [35,46]. Furthermore, antiviral activities have been reported against herpes simplex viruses (HSV-1 and HSV-2), polio virus (PV3), measles virus, and influenza virus [41,66]. Immunomodulatory and anticancer properties of enterocins have not been widely explored but may also be of potential interest [67,68,69].

In this study, we identified known bacteriocins, natural variants of known bacteriocins, and potentially new bacteriocins distributed among different enterococcal species. The potency and spectrum of bacteriocins against important pathogens vary according to the peptide subclass [34,35,66,70]. Class I bacteriocins were identified in our in silico screening, with sactipeptides, new lanthipeptides I, lasso peptides, and thiopeptides being found in high numbers (Figure 1). Sactipeptides are produced mainly by Gram-positive organisms, and according to previous studies, the sactipeptides from *Bacillus subtilis* (subtilisin A) and *Bacillus thuringiensis* (Thuricin CD) have broad and narrow antimicrobial activity spectra, respectively [34,71]. A previous study also identified sactipeptide BGC in *Enterococcus mudtii* QU25 [36], similar to one found in this study. Lantibiotics and thiopeptides are most active against Gram-positive pathogens, including MRSA, VRE, and *Clostridium difficile* [23,34]. In contrast, most lasso peptides show activity against Gram-negative pathogens, e.g., bacteriocin MccJ25, which is active against some strains of *Escherichia coli* and *Salmonella* spp. [34].

The present study provides further evidence of the significant biodiversity of BGCs for class II, 19 bacteriocins, including five new putative bacteriocins (Figure 1 and Figure 2; Supplementary Table S4). Class II bacteriocins are of special interest as potential therapeutic agents and have been proposed on a larger scale production, whether in the food industry or in human health and veterinary medicine [72,73,74]. Because they consist of unmodified peptides, they do not require enzymes for their maturation and are small structures, less than 10 kDa [36,73], that may subject to low-cost production than other classes by chemical synthesis [73]. Complementing the recombinant technologies, chemical synthesis of bacteriocins may allow further molecular engineering for enhanced potency, improved pharmacological properties, increased stability and modified spectra of activity [73]. Class II bacteriocins and analogs thereof have been successfully prepared by chemical syntheses, such as aureocin A53 (AucA), durancin A5-11, enterocin CRL35, lactococcin MMFII, leucocin A, pediocin PA-1, curvacin A, lacticin Q (LnqQ), mesentericin Y105, and sakacin P [72,73,74].

In general, the class II bacteriocins are most active against Gram-positive pathogens, especially the class IIa bacteriocins, which are active against *L. monocytogenes* and other Gram-positive pathogens [33,34,72,75]. Enterocin SE-K4 and enterocin P were the most frequent class II bacteriocins in this study (Figure 1). Enterocin SE-K4 has been reported to exhibit antimicrobial activity against Gram-positive bacteria, *B. subtilis*, *Clostridium beijerinckii*, *E. faecium*, *E. faecalis*, and *L. monocytogenes* [40]. In contrast, enterocin P has a broad antimicrobial spectrum that includes activity against food-borne pathogens, *C. botulinum*, *C. perfringens*, *L. monocytogenes*, and *S. aureus* [64], as well as clinical strains, *L. monocytogenes*, *Salmonella* (S.) *typhi*, *Salmonella paratyphi* C, *Shigella dysenteriae*, vancomycin-resistant enterococci (VRE), and carbapenem-resistant *Pseudomonas aeruginosa* [75,76].

It is also important to highlight that class III bacteriocins were most common and widely distributed from wild marine animals and also included the unknown bacteriocin VI (Figure 1). Furthermore, three different enterolysin A sequences were verified among enterococci species, with two of them from *E. hirae* genomes that are reported for the first time in this species. Enterolysin A is a cell wall-degrading bacteriocin first reported to be produced by *E. faecalis* isolated from fish in Iceland [77]. Despite class III bacteriocins are large proteins (more than 10 kDa) and complex produced by chemical approaches [61], enterolysin A have been reported as broad-spectrum activity against pathogenic and nonpathogenic bacteria; acting on cleave the peptide bonds within the stem peptide as well as in the interpeptide bridge of Gram-positive bacterial cell walls [33,78].

In addition to bacteriocins, a wide variety of novel gene clusters encoding putative terpenes, NRPs, polyketides, and other active compounds have been uncovered by in silico analysis, creating new opportunities for drug development [23,24,49,79]. NRPs and terpenes have been reported with activity against several antibiotic-resistant strains [80,81,82,83,84,85]. A small library of predicted NRP peptides was chemically synthesized, based on the primary sequence of NRP clusters in the human microbiome, and a potent anti-MRSA (methicillin-resistant *Staphylococcus aureus*) peptide with a new mechanism of action, named humimycin, was identified [80]. The antitubercular agent levesquamide is a new polyketide-nonribosomal peptide (PK-NRP) hybrid of a marine natural product (BGC) identified and isolated from *Streptomyces* sp. [84]. Furthermore, the antibacterial activity of 33 free terpenes commonly found in essential oils was evaluated, with 16 compounds showing antimicrobial activity, including eugenol, which exhibited rapid bactericidal action against *Salmonella enterica* serovar *Typhimurium*. Further, terpineol showed excellent bactericidal activity against *S. aureus* strains, and carveol, citronellol, and geraniol were rapidly bactericidal for *E. coli* [81]. In this study, we also found terpene biosynthesis-related clusters in *E. casseliflavus*, *E. hirae*, and *E. mundtii* species. Terpenes are secondary metabolites found in plants, bacteria, and fungi and have been shown to act as antibiotics, hormones, flavor or odor constituents, and pigments [86,87,88]. Beukers and collaborators [89] also identified putative genes or operons involved in terpene synthesis in *E. hirae*, *E. villorum*, *E. gallinarum*, *E. durans*, and *E. casseliflavus* strains isolated from bovine feces. The role of terpenes in enterococcal biology, including their possible involvement as bacteriocins, remains unclear [89].

Previous studies have examined the probiotic potential of enterococci from the marine environment [43,90,91]. Marine probiont strains have been used in finfish aquaculture due to their health beneficial effect and low potential to transfer antibiotic resistance genes to pathogens through horizontal gene transfer [92]. The potential of 13 enterococci isolated from wild seals was evaluated in a previous study from our group, and five (36.46%) showed activity against *L. monocytogenes* ATCC 35152 in the double-agar layer test, and one of them should be a good candidate for probiotic application [43]. In the present study, genome screening for bacteriocins highlighted potential probiotic enterococcal strains lacking known virulence or resistance traits (Figure 5A, B). In particular, the *E. avium* (L8) genome contained gene clusters for bicereucin BsjA1 and BsjA2, enterocin NKR-5-3D, mundticin AT06, and unknown bacteriocin I; and the *E. mundtii* genome (MP7-18) encoded sacpeptide and mundticin AT06 variants. Members of the genus *Enterococcus* have not yet obtained the status of generally recognized as safe (GRAS), although some are already being used as probiotics and in the production of animal food additives to prevent diseases or to improve growth [93,94]. New regulations for probiotics that distinguish between safe and potentially harmful strains are needed [35]. The application of genomic approaches in probiotic research would improve the understanding of the molecular mechanisms that endow the genera with safe and favorable traits [95].

Host-associated microbes are a rich source of factors that regulate community structure in a manner compatible with host health [96,97]. Our findings show a considerable novelty of biosynthetic pathways to be found by exploring the genomes of wild marine-animals-associated microbes in remote ecologies with the potential to shape host-associated microbial population structures. The novel compounds and natural bacteriocin variants were discovered to provide the first leads for deriving new approaches for managing human-microbe interactions in health and disease. Besides, this data will inform and broaden the limits of known structural variation, knowledge of how structure relates to activity, and synthetic biology. In this context, as a perspective for further studies, the data generated here may be associated with recombinant technologies, chemical synthesis, molecular engineering, and other strategies to increase the biological potency, stability, and pharmacological properties in order to guarantee or modify the antimicrobial activity. Therefore, our results may contribute to promote the future development of bacteriocin-based drugs for potential use in managing animal and human health and as food preservatives.

## 4. Materials and Methods

### 4.1. Bacterial Strains

Twenty-two enterococci strains previously described [26,98,99] were evaluated in the present study. Briefly, the collection includes *Enterococcus* species isolated from fecal samples (cloacal/anal swabs or intestinal content) collected from 17 wild marine animals. These animals, including sea turtles (*n* = 3), seabirds (*n* = 8), and marine mammals (*n* = 6), were found along the North Coast of Rio Grande do Sul, Southern Brazil, from Torres Beach (29°21′32.2′′ S; 49°44′10.3′′ W) to Dunas Altas Beach, Palmares do Sul (30°23′58.75′′ S; 50°17′24.73′′ W), between July 2012 and April 2014 (Table 1). The enterococci collection was stored frozen at −20 °C in skim milk supplemented with 20% glycerol, and cultures were routinely grown in brain heart infusion (BHI) at 37 °C for 18 h.

### 4.2. Genomic DNA Preparation, High-Throughput Sequencing, Assembly, and Annotation

The *Enterococcus* spp. strains were grown in BHI at 37 °C for 18 h. Genomic DNA was extracted using a commercial kit (QIAGEN DNeasy Blood & Tissue Kit, San Luis, MO, USA). Manufacturer instructions were followed with minor modification, namely, the addition of 50 μL of lysozyme (50 mg/mL) and 10 μL mutanolysin (2500 U/mL, Sigma-Aldrich, Germantown, MD, USA) for 30 min at 37 °C before the addition of 20 μL proteinase K (20 mg/mL). Extracted DNA was quantified using the Qubit double-stranded DNA (dsDNA) high-sensitivity (HS) assay kit (Life Technologies, Carlsbad, CA, USA). Libraries for genome sequencing were prepared using the Nextera XT DNA kit and index primers (Illumina), and reads were generated by HiSeq/MiSeq reagent kit version 2 with 250 cycles on an Illumina HiSeq/Miseq platforms. Reads were subjected to de novo assembly using the CLC genomics workbench v8.0.3, and open reading frames (ORFs) were predicted using the NCBI Prokaryotic Annotation Pipeline—PGAP [100]. The enterococci species assignment was confirmed by pairwise comparison of their average nucleotide identity (ANI) using JSpeciesWS [101] and the following reference genomes available from GenBank (https://www.ncbi.nlm.nih.gov (accessed on 15 December 2020): *Enterococcus avium* ATCC 14025; *Enterococcus casseliflavus* ATCC 12755; *Enterococcus faecalis* ATCC 19433; *Enterococcus faecium* Aus0004 (Clade A1); *Enterococcus faecium* EnGen0007 (Clade A2); *Enterococcus faecium* Com12 (Clade B); *Enterococcus hirae* ATCC 9790; *Enterococcus lactis* KCTC 21015; *Enterococcus mundtii* ATCC 882. The GenBank accession number of reference strains is presented in Supplementary Table S2.

### 4.3. Genome Mining for Antimicrobial Compounds

Putative biosynthetic gene clusters (BGCs) were predicted using *anti*SMASH (antibiotics and Secondary Metabolite Analysis Shell 5.0) [57] and Bagel4 (bacteriocins and RiPP—Ribosomally synthesized and Post-translationally modified Peptides) [58] using the default parameters. The bacteriocin classification is in accordance with previous proposals for enterococci [33] and lactic acid bacteria [36] that accommodate the novel subclasses that are appearing over the last years, based on the biosynthesis mechanism and biological activity.

### 4.4. Phylogenetic Analysis

Amino acid sequences corresponding to bacteriocin genes (class II and class III) found in this work, along with reference sequences identified by AntiSMASH 5.0 [57] and Bagel4 [58], and Uniprot databases were aligned using MAFFT [102]. Guidance2 [103] was used to filter unreliable positions and generate a mega alignment encompassing 5 alternative alignments for the sequences. The mega alignment was used to infer the evolutionary history of these proteins by using the Maximum Likelihood method, based on the VT model [104]. A discrete Gamma distribution was used to model evolutionary rate differences among sites, and the rate variation model allowed for some sites to be evolutionarily invariable [105]. Significance was assessed via aLRT [106]. All evolutionary analyses were conducted in PhyML 3.0 [107]. Tree visualization and annotation were performed on Interactive Tree Of Life (iTOL) v [108].

### 4.5. Molecular Modeling

The structural modeling of unknown bacteriocins (I, II, III, IV, and VI) was performed using the I-TASSER package [62,63] since they were not suitable for traditional comparative modeling, requiring a combination of fragment and ab initio model building. UCSF Chimera [109] was used to visualize and edit the new bacteriocin structural models. Physico-chemical parameters were calculated with ProtParam [110].

### 4.6. Potential Virulence Markers

The comprehensive antibiotic resistance database (CARD/RGI-2017) [111] and Resfinder 3.2 [112] were used to identify antimicrobial resistance genes with default parameters and identification threshold of 60% identity over a length of 60% coverage, respectively. Virulence genes were predicted using VirulenceFinder [113], with a threshold of 85% identity over a length of 60%.

### 4.7. Figures Design

Figures were designed using D3 (or D3.js, a JavaScript library for visualizing data using web standards) [114], R software (R Development Core Team, 2019) [115], and Adobe Illustrator.

## 5. Conclusions

Our findings show that there is a considerable novelty to be found through exploring the genomes of host-associated microbes from animals in remote ecologies for biosynthetic pathways with the potential to shape host-associated microbial population structures. The novel compounds and natural bacteriocin variants discovered provide first leads for the derivation of new approaches for managing human-microbe interactions in health and disease.

## Figures and Tables

**Table 1:** The description of the origin of enterococci genomes associated with wild marine animals.

Animal	Common Name	Scientific Name	Age ^1^	Code ^2^	Collection Date	Location	Enterococci Genomes ^3^	Species Identification (ANI ^5^)	Collection from
Sea turtles	green turtle	*Chelonia mydas*	Y	2	29-May-13	Cidreira	GT3-2	*E. faecalis* (98.38)	Prichula et al. (2016) Prichula et al. (2020)
	green turtle	*Chelonia mydas*	Y	1	25-Apr-14	Tramandaií	GT6-1	*E. faecalis* (98.53)	
	hawksbill turtle	*Eretmochelys imbricata*	Y	1	23-Dec-12	Tramandaií	HT1-3	*E. casseliflavus* (98.56)	
Seabirds	Magellanic penguin	*Spheniscus magellanicus*	Y	1	2-Nov-12	Cidreira	MP1-1	*E. hirae* (98.36)	
							MP1-2	*E. hirae* (98.37)	
							MP1-4	*E. hirae* (99.34)	
							MP1-5	*E. hirae* (98.68)	
	Magellanic penguin	*Spheniscus magellanicus*	Y	1	13-Nov-12	Xangri-laí	MP2-6 ^4^	*E. faecalis* (98.55)	
	Magellanic penguin	*Spheniscus magellanicus*	Y	2	27-Jul-13	Cidreira	MP5-1 ^4^	*E. faecalis* (98.54)	
	Magellanic penguin	*Spheniscus magellanicus*	Y	1	19-Sep-13	Imbeí	MP7-18	*E. mundtii* (97.04)	
	Magellanic penguin	*Spheniscus magellanicus*	Y	1	14-Oct-13	Cidreira	MP8-1 ^4^	*E. faecalis* (98.52)	
							MP8-17 ^4^	*E. faecalis* (98.67)	
	Magellanic penguin	*Spheniscus magellanicus*	Y	1	16-Oct-13	Cidreira	MP9-10 ^4^	*E. faecalis* (98.52)	
	Magellanic penguin	*Spheniscus magellanicus*	Y	1	23-Dec-13	Torres	MP10-1	*E. lactis* (98.92)	
	snowy-crowned tern	*Sterna trudeaui*	A	2	4-Dec-13	Arroio do Sal	ST1-20	*E. faecalis* (98.63)	
Marine Mammals	dwarf minke whale	*Balaenoptera acutorostrata*	Y	2	21-Jun-13	Tramandaií	DMW1-1	*E. hirae* (98.09)	
	Risso’s dolphin	*Grampus griseus*	A	2	4-Jul-13	Balneaírio Pinhal	RD1-1	*E. faecalis* (98.71)	
	South American fur seal	*Arctocephalus australis*	-	2	2-Aug-12	Torres	B9	*E. faecalis* (98.81)	Santestevan et al. (2015)
	South American fur seal	*Arctocephalus australis*	A	2	2-Aug-12	Xangri-laí	C7	*E. hirae* (98.67)	
	South American fur seal	*Arctocephalus australis*	A	2	12-Jul-12	Palmares do Sul	J2	*E. casseliflavus* (98.56)	
							J4	*E. casseliflavus* (98.57)	
	South American fur seal	*Arctocephalus australis*	-	2	21-Jul-12	Tramandaií	L8	*E. avium* (98.06)	

^1^ Age of the animals: A: adult; Y: young. ^2^ Code based on Geraci and Lounsbury (2005). ^3^ Strains were sequenced in this study. GT—green turtle; HT—hawksbill turtle; MP—Magellanic penguin; ST—snowy-crowned tern; DMW—dwarf minke whale; RD—Risso’s dolphin, and B, C, J or L—South American fur seal. ^4^ Genomes sequenced in a previous study (Prichula et al., 2020). ^5^ The enterococci species were confirmed by pairwise comparison of their average nucleotide identity (ANI) using as reference the following genomes: *Enterococcus avium* ATCC14025; *Enterococcus casseliflavus* ATCC12755; *Enterococcus faecalis* ATCC19433; *Enterococcus hirae* ATCC 9790; *Enterococcus lactis* KCTC 21015; *Enterococcus mundtii* ATCC 882.

## Data Availability

The novel genome sequences were deposited at DDBJ/ENA/GenBank as whole-genome shotgun projects under the accession numbers according to Table S1.

## Supplementary Materials

The following are available online at https://www.mdpi.com/article/10.3390/md19060328/s1, Table S1: Sequencing statistics, genome sizes, fold coverage, G+C content, of the *Enterococcus* spp. sequenced. Table S2: Reference genomes used to confirm the enterococci species. Table S3: Putative antimicrobial compounds biosynthesis gene clusters (BGCs) data predicted with antiSMASH5 and Bagel4 software. Table S4: Class I, class II, and class III unknown bacteriocins BGCs data that were not previously identified in antiSMASH5 and Bagel4 databases. Table S5: Class II and class III bacteriocin sequences predicted with antiSMASH5 and Bagel4 software. Table S6: Reference sequences from Bagel4 and Uniprot databases. Figure S1: The alignment of putative enterolysin A (class III) sequences (first branch) from *E. hirae* genomes using Clustal Omega software. Figure S2: The alignment of putative enterolysin A (class III) sequences (second branch) from *E. hirae* genomes using Clustal Omega software. Figure S3: The alignment of putative enterolysin A (class III) sequences (third branch) from *E. faecalis* genomes using Clustal Omega software. Figure S4: The alignment of four different enterolysin A (class III) and three different references (Bagel 62.3: *E. faecalis* LMG 2333; Bagel 63.3: *E. faecalis*; and Bagel 64.3: *Lactobacillus acidophilus*) using Clustal Omega software. Figure S5: The alignment of putative propionicin SM1 (class III) and reference sequence using Clustal Omega software. Figure S6: The alignment of putative Class IIa bacteriocins and reference sequences using Clustal Omega software. Figure S7: The alignment of putative class IIb bacteriocins and reference sequences using Clustal Omega software. Figure S8: The alignment of putative class II circular bacteriocin carnocyclin A and reference sequence using Clustal Omega software. Figure S9: The alignment of putative class II leaderless bacteriocin enterocin EJ97 and reference sequence using Clustal Omega software. Figure S10: The alignment of putative class II other bacteriocins and reference sequences using Clustal Omega software.
