# Supplementary material for: Genome Mining for Antimicrobial Compounds in Wild Marine Animals-Associated Enterococci
Source: Mar Drugs. 2021 Jun 6;19(6):328. doi: 10.3390/md19060328 (PMC8229437; doi:10.3390/md19060328)
Supplement: Supplementary file 1 [file marinedrugs-19-00328-s001.zip › marinedrugs-1226398-supplementary.pdf]

Supplementary Material

Genome mining for antimicrobial compounds in wild marine animals-associated enterococci

Janira Prichula, Muriel Primon-Barros, Romeu C. Z. Luz, Ícaro M. S. Castro, Thiago G. S. Paim, Maurício Tavares, Rodrigo Ligabue-Braun, Pedro A. d'Azevedo, Jeverson Frazzon, Ana P. G. Frazzon, Adriana Seixas and Michael S. Gilmore

Table S1: Sequencing statistics, genome sizes, fold coverage, G+C content, of the *Enterococcus* spp. sequenced.

GENOME	SEQUENCING TECNOLOGY	TOTAL BASES	SCAFFOLDS	GENOME SIZE (bp)	FOLD COVERAGE	G+C CONTENT (%)	GENBANK ACESSION #
**L8**	HiSeq	610359248	106	4,472,513	136x	39.2	VODK00000000
**HT1-3**	MiSeq	1375243158	21	3,884,582	354x	42.2	VODL00000000
**J2**	MiSeq	1306790762	32	3,812,258	342x	42.4	VODM00000000
**J4**	HiSeq	595411311	25	3,804,228	156x	42.3	VODN00000000
**B9**	MiSeq	434863771	15	2,720,842	159x	37.6	VODO00000000
**GT3-2**	MiSeq	714397735	51	3,097,641	230x	36.9	VODQ00000000
**GT6-1**	MiSeq	946754283	31	3,146,532	300x	37.1	VODT00000000
**MP2-6**[1]	HiSeq	641753007	16	2,744,259	233x	37.5	SJAU00000000
**MP5-1**[1]	HiSeq	663885394	40	3,058,640	217x	37.2	SMGV00000000
**MP8-1**[1]	HiSeq	453913397	55	3,137,577	144x	37.1	SMHA00000000
**MP8-17**[1]	HiSeq	512337670	12	2,742,168	186x	37.6	SJAV00000000
**MP9-10**[1]	HiSeq	432160424	45	3,064,363	141x	37.1	SMGY00000000
**RD1-1**	MiSeq	364892081	38	2,809,475	129x	37.6	VODX00000000
**ST1-20**	MiSeq	852868609	13	2,966,276	287x	37,3	VODY00000000
**MP10-1**	HiSeq	203305237	61	2,624,766	77x	38.2	VOEA00000000
**C7**	HiSeq	407008222	108	3,105,395	131x	36.6	VOEB00000000
**DMW1-1**	MiSeq	694005444	63	3,081,981	225x	36.3	VOEC00000000
**MP1-1**	MiSeq	872692755	11	2,929,511	297x	36.8	VOED00000000
**MP1-2**	MiSeq	458143673	36	3,078,537	148x	36.6	VOEE00000000
**MP1-4**	MiSeq	1148198824	22	2,624,579	437x	37.0	VOEF00000000
**MP1-5**	MiSeq	1534450898	17	2,899,878	529x	36.6	VOEG00000000
**MP7-18**	MiSeq	928652895	30	3,156,314	294x	38.3	VOEH00000000

[1]Genomes from previously study (Prichula et al., 2020).

Table S2: Reference genomes used to confirm the enterococci species.

REFERENCE STRAINS	REFSEQ ASSEMBLY ACCESSION
Enterococcus avium ATCC 14025	GCF_000407245.1
Enterococcus casseliflavus ATCC 12755	GCF_000191365.1
Enterococcus faecalis ATCC 19433	GCF_000392875.1
Enterococcus faecium Aus0004 - **Clade A1**[1]	GCF_000250945.1
Enterococcus faecium EnGen0007 - **Clade A2**[1]	GCF_000321845.1
Enterococcus faecium Com12 **- Clade B**[1]	GCF_000157635.1
Enterococcus hirae ATCC 9790	GCF_000393835.1
Enterococcus lactis KCTC 21015	GCF_015767715.1
Enterococcus mundtii ATCC 882	GCF_000393815.1

[1]Clade defined by Lebreton et al. [27].

Table S3: Putative antimicrobial compounds biosynthesis gene clusters (BGCs) data predicted with antiSMASH5 and Bagel4 software.

SPECIES	GENOME	SOFTWARE	BCGs[1] (73)	CONTIG	START	END	LENGTH	CONTENT/BLAST HIT	TYPE	MODSYSTEM	GENES (88)
E. avium	**L8**	AntiSMASH5/Bagel4	1	14	95756	115756	20000	259.1. Bicereucin_BsjA2	I	LanM	6
								258.1. Bicereucin_BsjA1	I	LanM	
								258.1. Bicereucin_BsjA1	I	LanM	
			1	55	83021	106372	23351	155.2. Mundticin_AT06	II	-	
								**Unknown Bacteriocin I**	II	-	
								89.2. Enterocin_NKR-5-3D	II	-	
E. casseliflavus	**HT1-3**	AntiSMASH5	1	2	194573	215496	20923	**Terpene**	Terpene	-	3
		Bagel4	1	10	8465	29776	21311	Sactipeptide	I	Sac_1	
			1	13	6878	27208	20330	186.2. Propionicin_SM1	III	-	
	**J2**	AntiSMASH5	1	8	13922	34845	20923	**Terpene**	Terpene	-	2
		Bagel4	1	15	13793	35104	21311	Sactipeptide	I	Sac_1	
	**J4**	AntiSMASH5	1	10	13922	34845	20923	**Terpene**	Terpene	-	2
		Bagel4	1	19	87107	108418	21311	Sactipeptide	I	Sac_1	
E. faecalis	**B9**	Bagel4	1	1	316232	336994	20762	63.3. Enterolysin_A	III	-	3
		AntiSMASH5/Bagel4	1	1	35594	55594	20000	Lasso peptide	I	LasB	
			1	7	137132	157132	20000	Thiopeptide	I	-	
	**GT3-2**	AntiSMASH5	1	51	1	1206	1205	95.2. Enterocin_SE-K4	II	-	5
		Bagel4	1	23	3170	16950	13780	109.2. Enterocin_96	II	-	
			1	32	2126	22888	20762	63.3. Enterolysin_A	III	-	
		AntiSMASH5/Bagel4	1	8	1	8987	8986	96.2. Enterocin_X_chain_alpha	II	Two components	
								97.2. Enterocin_X_chain_beta	II	Two components	
	**GT6-1**	Bagel4	1	2	0	11012	11012	95.2. Enterocin_SE-K4	II	-	9
		AntiSMASH5/Bagel4	1	18	95700	120409	24709	**Unknown Bacteriocin III**	II	-	
								**Unknown Bacteriocin IV**	III	-	
								209.2. Sakacin_Q	II	-	
								96.2. Enterocin_X_chain_alpha	II	Two components	
								97.2. Enterocin_X_chain_beta	II	Two components	
								**Unknown Bacteriocin V**	II	-	
								**Unknown Bacteriocin VI**	II	-	
			1	24	17857	38022	20165	148.1. Carnocyclin_A	II	Head to tail cyclized peptide	
	**MP2-6**	Bagel4	1	5	166490	188332	21842	Sactipeptides	I	Sac_1	1
	**MP5-1**	Bagel4	1	2	204053	224815	20762	63.3. Enterolysin_A	III	-	3
		AntiSMASH5/Bagel4	1	9	231467	251467	20000	Lasso peptide	I	LasB	
			1	13	49895	69895	20000	Thiopeptide	I	-	
	**MP8-1**	Bagel4	1	8	260924	281452	20528	63.3. Enterolysin_A	III	-	4
		AntiSMASH5/Bagel4	1	15	80933	100933	20000	Lasso peptide	I	LasB	
			1	20	1	19297	19296	16.2. Bacteriocin_31	II	-	
			1	21	86270	106270	20000	Thiopeptide	I	-	
	**MP8-17**	Bagel4	1	11	0	15058	15058	225.2. UviB	II	-	3
		AntiSMASH5/Bagel4	1	6	197081	219659	22578	159.1. Carnolysin	I	LanM	
								259.1. Bicereucin_BsjA2	I	LanM	
	**MP9-10**	Bagel4	1	17	93269	113797	20528	63.3. Enterolysin_A	III	-	3
		AntiSMASH5/Bagel4	1	11	85910	105910	20000	Lasso peptide	I	LasB	
			1	15	86267	106267	20000	Thiopeptide	I	-	
	**RD1-1**	Bagel4	1	16	4436	25108	20672	63.3. Enterolysin_A	III	-	1

	**ST1-20**	Bagel4	1	1	68224	88983	20759	63.3. Enterolysin_A	III	-	6
			1	6	676706	697468	20762	63.3. Enterolysin_A	III	-	
		AntiSMASH5/Bagel4	1	2	72260	93085	20825	16.2. Bacteriocin_31	II	-	
								95.2. Enterocin_SE-K4	II	-	
			1	2	95195	115273	20078	95.2. Enterocin_SE-K4	II	-	
								95.2. Enterocin_SE-K4	II	-	
E. lactis	**MP10-1**	AntiSMASH5	1	33	50740	88050	37310	**NRPs**	NRPS	-	3
			1	47	1	9228	9227	**NRPs**	NRPS	-	
		Bagel4	1	15	0	8463	8463	76.2. Enterocin_EJ97	II	-	
E. hirae	**C7**	AntiSMASH5	1	2	2629	23498	20869	**Terpene**	Terpene	-	7
		Bagel4	1	7	1	12492	12491	63.3. Enterolysin_A	II	-	
			1	8	79475	99475	20000	Sactipeptide	I	Sac_1	
			1	35	4181	24571	20390	63.3. Enterolysin_A	III	-	
			1	37	12290	27873	15583	63.3. Enterolysin_A	III	-	
		AntiSMASH5/Bagel4	1	2	42323	62323	20000	**Unknown Lanthipeptide I**	I	LanM	
			1	51	1	12205	12204	91.2. Enterocin_P	II	-	
	**DMW1-1**	Antismash	1	22	66142	87011	20869	**Terpene**	Terpene	-	8
			1	28	1	6799	6798	Uniprot_Q3XXB3_lactococcin 972	II	-	
		Bagel4	1	1	172226	192226	20000	Sactipeptide	I	Sac_1	
			1	29	177893	198163	20270	63.3. Enterolysin_A	III	-	
		AntiSMASH5/Bagel4	1	4	0	25491	25491	109.2. Enterocin_96	II	-	
								91.2. Enterocin_P	II	-	
			1	11	232348	252513	20165	**Unknown Bacteriocin II**	II	-	
			1	22	105800	125800	20000	**Unknown Lanthipeptide I**	I	LanM	
	**MP1-1**	AntiSMASH5	1	1	83767	104636	20869	**Terpene**	Terpene	-	4
		Bagel4	1	1	44978	64978	20000	**Unknown Lanthipeptide II**	I	LanM	
			1	3	502880	522880	20000	Sactipeptide	I	Sac_1	
			1	7	478	20766	20288	63.3. Enterolysin_A	III	-	
	**MP1-2**	AntiSMASH5	1	5	83814	96460	12646	**Terpene**	Terpene	-	4
		Bagel4	1	11	189098	209211	20113	63.3. Enterolysin_A	III	-	
			1	24	1	3422	3421	63.3. Enterolysin_A	III	-	
		AntiSMASH5/Bagel4	1	5	45017	65017	20000	**Unknown Lanthipeptide I**	I	LanM	
	**MP1-4**	AntiSMASH5	1	7	83867	104736	20869	**Terpene**	Terpene	-	4
		Bagel4	1	9	0	20034	20034	63.3. Enterolysin_A	III	-	
			1	12	165266	188809	23543	Sactipeptide	I	Sac_1	
		AntiSMASH5/Bagel4	1	7	45077	65077	20000	**Unknown Lanthipeptide I**	I	LanM	
	**MP1-5**	AntiSMASH5	1	10	84401	105270	20869	**Terpene**	Terpene	-	5
		Bagel4	1	15	1	12320	12319	63.3. Enterolysin_A	III	-	
		AntiSMASH5/Bagel4	1	4	894743	907731	12988	91.2. Enterocin_P	II	-	
								22.2. Bacteriocin_T8	II	-	
			1	10	45467	65467	20000	**Unknown Lanthipeptide I**	I	LanM	
E. mundtii	**MP7-18**	Antismash	1	11	91009	111905	20896	**Terpene**	Terpene	-	3
		Bagel4	1	5	47021	67021	20000	Sactipeptide	I	Sac_1	
		Antismash/Bagel4	1	10	25005	45176	20171	155.2. Mundticin_AT06	II	-	

[1] Number of biosynthetic genes clusters (BGCs) for antimicrobial compounds, including two non-ribosomal peptides – NRPs (red color), 10 terpenes synthesis routes (green color), and 61 bacteriocins involved genes (black color).

Table S4: Class I, class II, and class III unknown bacteriocins BGCs data that were not previously identified in antiSMASH5 and Bagel4 databases.

CLASS	BACTERIOCIN	GENOME	REGION	ORF	GENE START	GENE END	GENE STRAND	REAL START	REAL END	REGION SIZE
I	Unknown Lanthipeptide I	C7	Contig_2	orf00014	8122	11025	+	50445	53348	20000
		DMW1-1	Contig_22	orf00013	8121	11024	+	113921	116824	20000
		MP1-2	Contig_5	orf00016	8977	11880	-	53994	56897	20000
		MP1-4	Contig_7	orf00017	8976	11879	-	54053	56956	20000
		MP1-5	Contig_10	orf00016	8977	11880	-	54444	57347	20000
	Unknown Lanthipeptide II	MP1-1	Contig_1	orf00016	10219	11898	-	55197	56876	20000
II	Unknown Bacteriocin I	L8	Contig_55	orf00018	10882	11085	+	93903	94106	23351
	Unknown Bacteriocin II	DMW1-1	Contig_11	orf00025	10165	10347	-	242166	242348	20165
	Unknown Bacteriocin III	GT6-1	Contig_18	orf00018	7067	7273	+	102767	102973	24709
	Unknown Bacteriocin IV	GT6-1	Contig_18	orf00025	8244	8450	+	103944	104150	24709
	Unknown Bacteriocin V	GT6-1	Contig_18	orf00050	17955	18185	+	113655	113885	24709
III	Unknown Bacteriocin VI	GT6-1	Contig_18	orf00060	20237	20914	-	115937	116614	24709

Table S5: Class II and class III bacteriocin sequences predicted with antiSMASH5 and Bagel4 software.

GENOME	BACTERIOCIN	CLASS	AA SEQUENCE
MP8-1	Bacteriocin 31	IIa	MKKKLVKGLVICGMIGIGFTALGTNVEAATYYGNGLYCNKQKCWVDWNKASREIGKIIVNGWVQHGPWAPR
ST1-20	Bacteriocin 31	IIa	MKKKLVKALVICGMIGLGFTSLGTNAEAATYYGNGLYCNKQKCSVDWNKASREIGKIIVNGWVQHGPWAPR
MP1-5	Bacteriocin T8	IIa	MIMKKKVLKHCVILRILGTCLAGIGTGIDVDAATYYGNVLYCNKEKCWVNWGQSWSEGLKRWGDNLFGSFIGGR
GT6-1	Carnocyclin A	II (circular)	MYKQGGIFMVYDLVAYGIAQGVAEKIVGLINAGLTVGSIISIIGGATAGLAGVFAAVKAAIAKQGIKKAIQL
GT3-2	Enterocin 96	II (others)	VERTKGDNTMLNKKLLENGVVNAVTIDELDAQFGGMSKRDCNLMKACCAGQAVTYAIHSLLNRLGGDSSDPAGCNDIVRKYCK
DMW1-1	Enterocin 96	II (others)	MINKKLFDSGIVNPVTIEDLDNQFGGPKRQCSLMKACCVGQAVTYVIHNPLNQMGRDSSDPEGCNAIVRKYC
MP10-1	Enterocin EJ97	II (leaderless)	VISMKFKFNPTGTIVKKLTQYEIAWFKNKHGYYPWEIPRC
L8	Enterocin NKR-5-3D	II (others)	MLKKKLVIKEEQKKIKGGTPGGFDYLTAGPHAAKGILNAIKNYFK
C7	Enterocin P	IIa	VTNFGTKVDAATRSYGNGVYCNNSKCWVNWGEAKENIAGIVISGWAYGLAGMGH
DMW1-1	Enterocin P	IIa	MRKKLFSLALIGTFGLAVTNFGTKVDAATRSYGNVVYCNNSKCWVNWEEAKENIAGIIISGWASGLAGMGH
MP1-5	Enterocin P	IIa	MRKKLFSLALIGTFGLAVTNFGTKVDAATRSYGNVVYCNNSKCWVNWEEAKENIAGIIISGWASGLAGMGH
GT3-2	Enterocin SE-K4	IIa	MIGIGFTALGTNVEAATYYGNGVYCNKQKCWVDWSRARSEIIDRGVKAYVNGFTKVLGGIGGR
GT6-1	Enterocin SE-K4	IIa	MKKKLVKGLVICGIIRIGFIALGTNIEAATYYGNGVYCNSKLGTSLVRRS
ST1-20**_orfblast_1**	Enterocin SE-K4	IIa	MKKKLVKGLVICGMIGIGFTALGMNVEAATYYGNGVYCNKQKCWVNWGQAWSEGVKRxxxxLFGSFSGGRI
ST1-20**_orf00012**	Enterocin SE-K4	IIa	MKKKLVKGLVICGMIGIGFTALGMNVEAATYYGNGVYCNKQKCWVNWGQAWSEGVKR
ST1-20**_orf00031**	Enterocin SE-K4	IIa	MKKKLVKGLVICGMIGIGFTALGTNVEAATYYGNGVYCNKQKCWVDWSRARSEIIDRGVKAYVNGFTKVLGGIGGR
GT3-2	Enterocin X chain alpha	IIb	MKKYKVLTEKEMKQTVGGSNDGFWERVGVGIGAGSKCYAHGGRVKGYGMIPPLCVAYGIGAAFKG
GT6-1	Enterocin X chain alpha	IIb	MQDYKELNEKEMKKTVGGSNDGFWERVGVGIGAGSKCYANGGSVKGYDMIPPLCVAYGVGAAFKG
GT3-2	Enterocin X chain beta	IIb	MIKKELTDKELKKINGGVFPVVPVIVGGVLTYLGKQAFEHSDQIVKGFKKGWNKY
GT6-1	Enterocin X chain beta	IIb	MIKKELTNKELKKINGGVFPVVPVIVGGVLTYLGKQAFEHADQIGKGFKKGWNKY
DMW1-1	Lactococcin 972	II (others)	MKKFLCLSALMGVLLTSGGIVSATEALNLDVPEDHTAIYGGGMEATEEGFSSSKLRYAAGGGDFNCGVNGFKVYANYYHARAKHSATAKNGRGG QVRSVQKAGVRAYATCNATLTGNTGWWNVY
L8	Mundticin AT06	IIa	MECVDMTRSKKLNLREMKNIVGGTYYGNGVSCNKKGCSVDWGKAISIIGNNSAANLATGGAAGWKS
MP7-18	Mundticin AT06	IIa	LKKLTAKEMSQVVGGKYYGNGVSCNKKGCSVDWGKAIGIIGNNSAANLATGGAAGWKS
GT6-1	Sakacin Q	II (others)	MQNVKELSSVEMQKTIGGAKWSKEQYLNTCVAGAYGAALSGAAKHWKLGPGALVGALGSEISYMSQNGCFNKNGA
MP8-17	UviB	II (others)	MKVGEILEEFVKGLLTNPEQISFAVLFVSLLFWVMKQNNDREQNYQKTIDKLADSLKDVESIKTTVEKINEKLN

L8	Unknow Bacteriocin I	II	MIKKDVLKKVDLKKVIGGGGASGTWLDSKTKACINGQAGGMLAGSPGGLGGIIIGGIGGAIAGGCFG
DMW1-1	Unknow Bacteriocin II	II	MKKLTAEEMKQVVGGRVHLSNNTKACINGQLGGMLTGSVGGIGGIILGGIAGAIAGGCFN
GT6-1	Unknow Bacteriocin III	II	MENFKELTVKEMQKISGGGWQTMSFTPNMECWNGILKTGNCRVKWDVVANQAVNNVTSAMIGGFGRGR
GT6-1	Unknow Bacteriocin IV	II	MTKFKELTVQEMKQISGGKHGKPIYFKDLPWAQQKCILSVAGGALIGTTTGGPLGALLGAGSQAWGCL
GT6-1	Unknow Bacteriocin V	II	MQNMKELTAKDTQQINGGGWSTPPGLSNIECKNGHLAVGNCRAKWGDISNGLVNQLVSCAVNGMYGGRCKQPGKFY
B9	Enterolysin A	III	MKNILLSILGVLSIVVSLAFSSYSVNAASNEWSWPLGKPYAGRYEEGQQFGNTAFNRGGTYFHDGFDFGSAIYGNGSVYAVHDGKILYAGWDPV<br>GGGSLGAFIVLQAGNTNVIYQEFSRNVGDIKVSTGQTVKKGQLIGKFTSSHLHLGMTKKEWRSAHSSWNKDDGTWFNPIPILQGGSTPTPPNPG<br>PKNFTTNVRYGLRVLGGSWLPEVTNFNNTNDGFAGYPNRQHDMLYIKVDKGQMKYRVHTAQSGWLPWVSKGDKSDTVNGAAGMPGQAIDGVQLN<br>YITPKGEKLSQAYYRSQTTKRSGWLKVSADNGSIPGLDSYAGIFGEPLDRLQIGISQSNPF
GT3-2	Enterolysin A	III	MKKILISVLGVLSIVVSMAFSSYSVNAASNNWSWPLGKPYAGRYEEGQQFGNTAFDRGGTYFHDGFDFGSAIYGNGSVYAVYDGKILYAGWDPV<br>GGGSLGAFIVLQAGDTNVIYQEFSRNVGDIKVSTGQTVKKGQLIGNFTSSHLHLGMTKKEWRAAHSSWNKDDGTWFNPIPILQGGSTPTPPNPG<br>PTNFTTNVHYGLHVLGGSWLGEVTNFNNTNDGFAGYPNRQHDMLYIKVDKGELKYRVHTAQSGWLAWVNKGNKNDTVNGVAGIQGQAIDGVQLN<br>YITPKGEKLSQAYYRSQTTKRSGWLKVSADNGSIPGLDSYAGIFGEPLDRLQIGISQSNPF
MP5-1	Enterolysin A	III	MKNILLSILGVLSIVVSLAFSSYSVNAASNEWSWPLGKPYAGRYEEGQQFGNTAFNRGGTYFHDGFDFGSAIYGNGSVYAVHDGKILYAGWDPV<br>GGGSLGAFIVLQAGNTNVIYQEFSRNVGDIKVSTGQTVKKGQLIGKFTSSHLHLGMTKKEWRSAHSSWNKDDGTWFNPIPILQGGSTPTPPNPG<br>PKNFTTNVRYGLRVLGGSWLPEVTNFNNTNDGFAGYPNRQHDMLYIKVDKGQMKYRVHTAQSGWLPWVSKGDKSDTVNGAAGMPGQAIDGVQLN<br>YITPKGEKLSQAYYRSQTTKRSGWLKVSADNGSIPGLDSYAGIFGEPLDRLQIGISQSNPF
MP8-1	Enterolysin A	III	MKNILLSILGVLSIVVSLAFSSYSVNAASNEWSWPLGKPYAGRYEEGQQFGNTAFNRGGTYFHDGFDFGSAIYGNGSVYAVHDGKILYAGWDPV<br>GGGSLGAFIVLQAGNTNVIYQEFSRNVGDIKVSTGQTVKKGQLIGKFTSSHLHLGMTKKEWRSAHSSWNKDDGTWFNPIPILQGGSTPTPPNPG<br>PKNFTTNVRYGLRVLGGSWLPEVTNFNNTNDGFAGYPNRQHDMLYIKVDKGQMKYRVHTAQSGWLPWVSKGDKSDTVNGAAGMPGQAIDGVQLN<br>YITPKGEKLSQAYYRSQTTKRSGWLKVSADNGSIPGLDSYAGIFGEPLDRLQIGISQSNPF
MP9-10	Enterolysin A	III	MKNILLSILGVLSIVVSLAFSSYSVNAASNEWSWPLGKPYAGRYEEGQQFGNTAFNRGGTYFHDGFDFGSAIYGNGSVYAVHDGKILYAGWDPV<br>GGGSLGAFIVLQAGNTNVIYQEFSRNVGDIKVSTGQTVKKGQLIGKFTSSHLHLGMTKKEWRSAHSSWNKDDGTWFNPIPILQGGSTPTPPNPG<br>PKNFTTNVRYGLRVLGGSWLPEVTNFNNTNDGFAGYPNRQHDMLYIKVDKGQMKYRVHTAQSGWLPWVSKGDKSDTVNGAAGMPGQAIDGVQLN<br>YITPKGEKLSQAYYRSQTTKRSGWLKVSADNGSIPGLDSYAGIFGEPLDRLQIGISQSNPF
RD1-1	Enterolysin A	III	MKNILLSILGVLSIVVSLAFSSYSVNAASNEWSWPLGKPYAGRYEEGQQFGNTAFNRGGTYFHDGFDFGSAIYGNGSVYAVHDGKILYAGWDPV<br>GGGSLGAFIVLQAGNTNVIYQEFSRNVGDIKVSTGQTVKKGQLIGKFTSSHLHLGMTKKEWRSAHSSWNKDDGTWFNPIPILQGGSTPTPPNPG<br>PKNFTTNVRYGLRVLGGSWLPEVTNFNNTNDGFAGYPNRQHDMLYIKVDKGQMKYRVHTAQSGWLPWVSKGDKSDTVNGAAGMPGQAIDGVQLN<br>YITPKGEKLSQAYYRSQTTKRSGWLKVSADNGSIPGLDSYAGIFGEPLDRLQIGISQSNPF
ST1-20_**orf00016**	Enterolysin A	III	VKKTLISVLGVLSIVVSMAFSSYSVNAASNDWSWPLGKPYAGRYEEGQQFGNTAFDRGGTYFHDGFDFGSAIYGNGSVYAVHDGKILYAGWDPV<br>GGGSLGAFIVLQAGDTNVIYQEFSRNVGDIKVSTGQTVKKGQLIGNFTSSHLHLGMTKKEWRAAHSSWNKDDGTWFNPIPILQGGSTPTPPNPG<br>PTNFTTNVHYGLHVLGGSWLGEVTNFNNTNDGFAGYPNRQHDMLYIKVDKGELKYRVHTAQSGWLAWVNKGNKNDTVNGVAGIQGQAIDGVQLN<br>YITSKGEKLSQAYYRSQTTKRSGWLKVSADNGSIPGLDSYAGIFGEPLDRLQIGISQSNPF
ST1-20_**orf00030**	Enterolysin_A	III	MKNILLSILGVLSIVVSLAFSSYSVNAASNEWSWPLGKPYAGRYEEGQQFGNTAFNRGGTYFHDGFDFGSAIYGNGSVYAVHDGKILYAGWDPV<br>GGGSLGAFIVLQAGNTNVIYQEFSRNVGDIKVSTGQTVKKGQLIGKFTSSHLHLGMTKKEWRSAHSSWNKDDGTWFNPIPILQGGSTPTPPNPG<br>PKNFTTNVRYGLRVLGGSWLPEVTNFNNTNDGFAGYPNRQHDMLYIKVDKGQMKYRVHTAQSGWLPWVSKGDKSDTVNGAAGMPGQAIDGVQLN<br>YITPKGEKLSQAYYRSQTTKRSGWLKVSADNGSIPGLDSYAGIFGEPLDRLQIGISQSNPF
**C7_orf00005**	Enterolysin A	III	MIESKERTTIMGQFETAKTIWDYLVSQGWSKTAVAGLLGNMQSESGIIADRWESDIVGNMSGGYGLVQWTPASKYINWANANGLNYKDVISQCK<br>RIEWEVANNQQFYNISMTFQQFKTSNKSPEELANIFIQYYERPANANQPARAQQARYWYQQLNRTKVVDWFNKHRGQITYSMTGSRNGTDGTAD<br>CSGSITQAVKDSSGIPYSYLYNTVTLGGYLQRCGYVLVLVGNSNASNLSQLKDEDIVLLSGGSSMADSGGAIGHTGVITGGGKNITSTCYYTQG<br>EKNTAIQELRFDKNYIVANGFHYYEVWRFSGGFNQSSSTGITNDPTPPSFSTNVHYSLRVLGGAWLGEITNFNNSDSNGFSGLPNHQHDMLYIK<br>VDKGTLRYRVHTMTSGWLDWVSKGDPNDMVNGCAGNPGEAIDGVQIYYTTPAGETYSQAYYRSQTTARADWLQTCCDDGTSIVGYDGWAGMFGE<br>PLDRLQIGIAKSNPLFTYSPGVNNGGSFSTNVHYGLRVLGGSWLGEITNFNDVDSNGFSGLPNNQHDMLYIKVDAGTIRYRVHTVKSGWLDWVS<br>KGDPNDMVNGCAGNPGEAIDGVQLYYTTPTGKTLSQAYYRSQTTARAGWLGVCCDDGTSIAGYDGWAGMFGEPLDRLQISIAASNQFFD
**C7_orf00014**	Enterolysin A	III	MSFLLFFLGIDDSDTGGSTAGGTEFNGVYTEDLPSYPEIKGVGNVPDEIAQLAVGSAVKYHLLPSVIISQWAYESEWGHSASAKNDNNFFGITW<br>FEGCPFPKGTARGVGGSEGGNYMKFPNKKSAFSYYGYMVASQTNFNACVGNKSPEQCLLTLGRGGYAAAGITMNSPYFTGCMSIIKSNNLTQYD<br>DFAIKKWKDFGGNTGGSVGGGWGWPFPDAGQGSFAGGQLFGKNPGGEFRENGFHDGLDFGSVDHPGNEIHAIHGGTVTYVGNPGISGLGACVIV<br>INDSGLNMVYQEFATSTSNAKVKVGDKVKLGDVIGIRDTEHLHLGITKKDWLQAESSAFTDDGTWLDPLKIITTGKY

C7_**orf00015**	Enterolysin A	III	MKWKLMVFGSGLILLLLPFFIMLSMIIQMIGSVGATEEISSYSEGMFTGEYTEDLPIFEEIKGRGPITDEHARYAVGAAVKYKLLPSVILSQLG<br>WESAYGQSYSGKNDNNYFGITWYSGCPYPKGSARGVGGSEGGNYMKFPDAKACYSYYGYMVATQSNFNACVGNKDPGQCLLILGRGGYAAAGIT<br>EGSAYYQGAMGIIKSYNLTEYDEFAIKKWGSIGSTSSGGSTGSLGGGWYNPFLGSSLERSSFLGGQLFGKNPGGEFRVNGFHNGLDFGSVDHPG<br>NEIHAVHAGTVVFAGNPSIAALGSCVIVIKDGDLSMVYQEFGSSPSNARVKVGDKVKAGQVIGIRDTAHLHLGFTKSDWYAAQASAFKNDGVWL<br>DPLPYLQQSGGRRED
DMW1-1	Enterolysin A	III	MIESKERTTIMGQFETAKTIWDYLVSQGWSKTAVAGLLGNMQSESGIIADRWESDIVGNMSGGYGLVQWTPASKYINWANANGLNYKDVISQCK<br>RIEWEVANNQQFYNISMTFQQFKTSNKSPEELANIFIQYYERPANANQPARAQQARYWYQQLNRTKVVDWFNKHRGQITYSMTGSRNGTDGTAD<br>CSGSITQAVKDSSGIPYSYLYNTVTLGGYLQRCGYVLVLVGNSNASNLSQLKDEDIVLLSGGSSMADSGGAIGHTGVITGGGKNITSTCYYTQG<br>EKNTAIQELRFDKNYIVANGFHYYEVWRFSGGFNQSSSTGMTNDPTPPSFSTNVHYSLRVLGGAWLGEITNFNNSDSNGFSGLPNHQHDMLYIK<br>VDKGTLRYRVHTMTSGWLDWVSKGDPNDMVNGCAGNPGEAIDGVQIYYTTPAGETYSQAYYRSQTTARADWLQTCCDDGTSIVGYDGWAGMFGE<br>PLDRLQIGIAKSNPLFTYSPGVNNGGSFSTNVHYGLRVLGGSWLGEITNFNDVDSNGFSGLPNNQHDMLYIKVDAGTIRYRVHTVKSGWLDWVS<br>KGDPNDMVNGCAGNPGEAIDGVQLYYTTPTGKTLSQAYYRSQTTARAGWLGVCCDDGTSIAGYDGWAGMFGEPLDRLQISIAASNQFFD
MP1-1	Enterolysin A	III	MIESKERTIIMGQFETAKTIWDYLVSQGWSKTAVAGLLGNMQSESGIIADRWESDIVGNMSGGYGLVQWTPASKYINWANANGLNYKDVISQCK<br>RIEWEVANNQQFYNISMTFQQFKTSNKSPEELANIFIQYYERPANANQPARAQQARYWYQQLNRTKVVDWFNKHRGQITYSMTGSRNGTDGTAD<br>CSGSITQAVKDSSGIPYSYLYNTVTLGGYLQRCGYVLVLVGNSNASNLSQLKDEDIVLLSGGSSMADSGGAIGHTGVITGGGKNITSTCYYTQG<br>EKNTAIQELRFDKNYIVANGFHYYEVWRFSGGFNQSSSTGITNDPTPPSFSTNVHYSLRVLGGAWLGEITNFNNSDSNGFSGLPNHQHDMLYIK<br>VDKGTLRYRVHTMTSGWLDWVSKGDPNDMVNGCAGNPGEAIDGVQIYYTTPAGETYSQAYYRSQTTARADWLQTCCDDGTSIVGYDGWAGMFGE<br>PLDRLQIGIAKSNPLFTYSPGVNNGGSFSTNVHYGLRVLGGSWLGEITNFNDVDSNGFSGLPNNQHDMLYIKVDAGTIRYRVHTVKSGWLDWVS<br>KGDPNDMVNGCAGNPGEAIDGVQLYYTTPTGKTLSQAYYRSQTTARAGWLGVCCDDGTSIAGYDGWAGMFGESLDRLQISIAASNQFFD
MP1-2_**orf00003**	Enterolysin A	III	MENQNESLIKQYVKRRAKRRLFLWLFGTSAGLITILITVFVTLFLILAAGSIDNSDSSSGGEAFTGEYSEGLPIYKEIKGRGQFSDEIAQYAVG<br>AAVKYKLLPSVILSQYGYESAFGTSASARNDLNYFGITWFDGCLFPKGTARGIGGIEGGWYMKFPNSKAAFSYYGFMVATQSNFNACVGNKSPG<br>ASLLILGRGGYAAAGITEDSAYYKNCMSIINKNKLTEYDEFAIKHWGEGGNNNGIITGEWTNPFPGSSLDKNSFSGGQLFGTNPGGEFRPNGFH<br>DGLDFGSVDHPGSEIHAVHGGKVVYVGNPGISGLGACVIVINYDGLNMVYQEFANSTGNSRVKVGDQVKVGQVIGIRDTAHLHLGFTRMDWRQA<br>QGHAFIDDGTWIDPLPFLNSSKK
MP1-2_**orf00023**	Enterolysin A	III	MIESKERTTIMGQFETAKTIWDYLVSQGWSKTAVAGLLGNMQSESGIIADRWESDIVGNMSGGYGLVQWTPASKYINWANANGLNYKDVISQCK<br>RIEWEVANNQQFYNISMTFQQFKTSNKSPEELANIFIQYYERPANANQPARAQQARYWYQQLNRTKVVDWFNKHRGQITYSMTGSRNGTDGTAD<br>CSGSITQAVKDSSGIPYSYLYNTVTLGGYLQRCGYVLVLVGNSNASNLSQLKDEDIVLLSGGSSMADSGGAIGHTGVITGGGKNITSTCYYTQG<br>EKNTAIQELRFDKNYIVANGFHYYEVWRFSGGFNQSSSTGMTNDPTPPSFSTNVHYSLRVLGGAWLGEITNFNNSDSNGFSGLPNHQHDMLYIK<br>VDKGTLRYRVHTMTSGWLDWVSKGDPNDMVNGCAGNPGEAIDGVQIYYTTPAGETYSQAYYRSQTTARADWLQTCCDDGTSIVGYDGWAGMFGE<br>PLDRLQIGIAKSNPLFTYSPGVNNGGSFSTNVHYGLRVLGGSWLGEITNFNDVDSNGFSGLPNNQHDMLYIKVDAGTIRYRVHTVKSGWLDWVS<br>KGDPNDMVNGCAGNPGEAIDGVQLYYTTPTGKTLSQAYYRSQTTARAGWLGVCCDDGTSIAGYDGWAGMFGEPLDRLQISIAASNQFFD
MP1-4	Enterolysin A	III	MIESKERTIIMGQFETAKTIWDYLVSQGWSKTAVAGLLGNMQSESGIIADRWESDIVGNMSGGYGLVQWTPASKYINWANANGLNYKDVISQCK<br>RIEWEVANNQQFYNISMTFQQFKTSNKSPEELANIFIQYYERPANANQPARAQQARYWYQQLNRTKVVDWFNKHRGQITYSMTGSRNGTDGTAD<br>CSGSITQAVKDSSGIPYSYLYNTVTLGGYLQRCGYVLVLVGNSNASNLSQLKDEDIVLLSGGSSMADSGGAIGHTGVITGGGKNITSTCYYTQG<br>EKNTAIQELRFDKNYIVANGFHYYEVWRFSGGFNQSSSTGMTNDPTPPSFSTNVHYSLRVLGGAWLGEITNFNNSDSNGFSGLPNHQHDMLYIK<br>VDKGTLRYRVHTMTSGWLDWVSKGDPNDMVNGCAGNPGEAIDGVQIYYTTPAGETYSQAYYRSQTTARADWLQTCCDDGTSIVGYDGWAGMFGE<br>PLDRLQIGIAKSNPLFTYSPGVNNGGSFSTNVHYGLRVLGGSWLGEITNFNDVDSNGFSGLPNNQHDMLYIKVDAGTIRYRVHTVKSGWLDWVS<br>KGDPNDMVNGCAGNPGEAIDGVQLYYTTPTGKTLSQAYYRSQTTARAGWLGVCCDDGTSIAGYDGWAGMFGEPLDRLQIGIAASNQFFD
MP1-5	Enterolysin A	III	MIESKERTTIMGQFETAKTIWDYLVSQGWSKTAVAGLLGNMQSESGIIADRWESDIVGNMSGGYGLVQWTPASKYINWANANGLNYKDVISQCK<br>RIEWEVANNQQFYNISMTFQQFKTSNKSPEELANIFIQYYERPANANQPARAQQARYWYQQLNRTKVVDWFNKHRGQITYSMTGSRNGTDGTAD<br>CSGSITQAVKDSSGIPYSYLYNTVTLGGYLQRCGYVLVLVGNSNASNLSQLKDEDIVLLSGGSSMADSGGAIGHTGVITGGGKNITSTCYYTQG<br>EKNTAIQELRFDKNYIVANGFHYYEVWRFSGGFNQSSSTGITNDPTPPSFSTNVHYSLRVLGGAWLGEITNFNNSDSNGFSGLPNHQHDMLYIK<br>VDKGTLRYRVHTMTSGWLDWVSKGDPNDMVNGCAGNPGEAIDGVQIYYTTPAGETYSQAYYRSQTTARADWLQTCCDDGTSIVGYDGWAGMFGE<br>PLDRLQIGIAKSNPLFTYSPGVNNGGSFSTNVHYGLRVLGGSWLGEITNFNDVDSNGFSGLPNNQHDMLYIKVDAGTIRYRVHTVKSGWLDWVS<br>KGDPNDMVNGCAGNPGEAIDGVQLYYTTPTGKALSQAYYRSQTTARAGWLGVCCDDGTSIAGYDGWAGMFGEPLDRLQIGIAASNQFFD
HT1-3	Propionicin SM1	III	MKKFIWSMLILGSVVGSTSIVSADSISNSEDTVELSPKQEQVQIAVENNDVLSDVSWALDYTQPVQIQALAKAVSDYSYSDYFSSVKWITRDGK<br>VSLSITPKAILTKNLPSGNGGAAHIGNAWNKLLAKHKNDKNWKNTNGMLDQYLCHAQWASGMKTPWNIEPWRPDVSYAATVAKACNP
GT6-1	Unknow Bacteriocin VI	III	MVPNIRKKAGDFMELQVSRKSKFFCLAMALLIALGMFISAGTSVYAAEVNNDISEEDKVILDNIDVNSFYSDANKGLNEFFSKAVSANPINGKL<br>ALNEIGAKDMFGEGIEYEAVVSFIEFFNSDNNFNELGRFEFRDSLKTLAQGNLPIQTRAGGALAKCAVEWAKNTFGVGISVAAFKSVLNTYGYA<br>KAAAWLAGKVASSTGRKAAAVLTLVWTAMTCAPIEAE

Table S6: Reference sequences from Bagel4 and Uniprot databases.

BACTERIOCINS	CLASS	AA SEQUENCES
**Bacteriocin 31** (Bagel: 16.2)	IIa	MKKKLVICGIIGIGFTALGTNVEAATYYGNGLYCNKQKCWVDWNKASREIGKIIVNGWVQHGPWAPR
**Bacteriocin T8** (Bagel: 22.2)	IIa	MKKKVLKHCVILGILGTCLAGIGTGIKVDAATYYGNGLYCNKEKCWVDWNQAKGEIGKIIVNGWVNHGPWAPRR
**Carnocyclin A** (Bagel: 148.1)	II (circular)	MLYELVAYGIAQGTAEKVVSLINAGLTVGSIISILGGVTVGLSGVFTAVKAAIAKQGIKKAIQL
**Enterocin 96** (Bagel: 109.2)	II (others)	MLNKKLLENGVVNAVTIDELDAQFGGMSKRDCNLMKACCAGQAVTYAIHSLLNRLGGDSSDPAGCNDIVRKYCK
**Enterocin EJ97** (Bagel: 76.2)	II (leaderless)	MLAKIKAMIKKFPNPYTLAAKLTTYEINWYKQQYGRYPWERPVA
**Enterocin NKR-5-3D** (Bagel: 89.2)	II (others)	MTNRKILPKEELKKIKGGTPGGIDFISGGPHVAQDVLNAIKNFFK
**Enterocin P** (Bagel: 91.2)	IIa	MRKKLFSLALIGIFGLVVTNFGTKVDAATRSYGNGVYCNNSKCWVNWGEAKENIAGIVISGWASGLAGMGH
**Enterocin SE-K4** (Bagel: 95.2)	IIa	MKKKLVKGLVICGMIGIGFTALGTNVEAATYYGNGVYCNKQKCWVDWSRARSEIIDRGVKAYVNGFTKVLGGIGGR
**Enterocin X chain alpha** (Bagel: 96.2)	IIb	MQNVKEVSVKEMKQIIGGSNDSLWYGVGQFMGKQANCITNHPVKHMIIPGYCLSKILG
**Enterocin X chain beta** (Bagel: 97.2)	IIb	MKKYNELSKKELLQIQGGIAPIIVAGLGYLVKDAWDHSDQIISGFKKGWNGGRRK
**Lactococcin 972** (Uniprot: Q3XXB3)	II (others)	MKKFLCLSALMGVLLTSGGIVSATEALNLDVPEDHTAIYGGGMEATEEGFSSSKLRYAAGGGDFNCGVNGFKVYANYYHARAKHSATAKN<br>GRGGQVRSVQKAGVRAYATCNATLTGNTGWWNVY
**Mundticin AT06** (Bagel: 155.2)	IIa	MKKLTAKEMSQVVGGKYYGNGVSCNKKGCSVDWGKAIGIIGNNSAANLATGGAAGWKS
**Sakacin Q** (Bagel: 209.2)	II (others)	MQNTKELSVVELQQILGGKRASFGKCVVGAAGLGAGVSGGLWGMAAGGIGGELAYMGANGCL
**UviB** (Bagel: 225.2)	II (others)	MDSELFKLMATQGAFAILFSYLLFYVLKENSKREDKYQNIIEELTELLPKIKEDVEDIKEKLNK
**Enterolysin A** (Bagel: 63.3)	III	MKNILLSILGVLSIVVSLAFSSYSVNAASNEWSWPLGKPYAGRYEEGQQFGNTAFNRGGTYFHDGFDFGSAIYGNGSVYAVHDGKILYAG<br>WDPVGGGSLGAFIVLQAGNTNVIYQEFSRNVGDIKVSTGQTVKKGQLIGKFTSSHLHLGMTKKEWRSAHSSWNKDDGTWFNPIPILQGGS<br>TPTPPNPGPKNFTTNVRYGLRVLGGSWLPEVTNFNNTNDGFAGYPNRQHDMLYIKVDKGQMKYRVHTAQSGWLPW
**Propionicin SM1** (Bagel: 186.2)	III	MNKTHKMATLVIAAILAAGMTAPTAYADSPGNTRITASEQSVLTQILGHKPTQTEYNRYVETYGSVPTEADINAYIEASESEGSSSQTAA<br>HDDSTSPGTSTEIYTQAAPARFSMFFLSGTWITRSGVVSLSLKPRKGGIGNEGDERTWKTVYDKFHNAGQWTRYKNNGVDASMKKQYMCH<br>FKYGMVKTPWNLEPHKKAADVSPVKCN

```
GENOME              BACTERIOCIN      AA SEQUENCE

C7_orf00015         Enterolysin A    ------------------MKWKLMVFG--SGLILLLLPFFIMLSMIIQMIGSVGATEEIS 40
C7_orf00014         Enterolysin A    ---------------------------------------MSFLLFFL---GIDDSDTGGS 18
MP1-2_orf00003      Enterolysin A    MENQNESLIKQYVKRRAKRRLFLWLFGTSAGLITILITVFVTLFLIL---AAGSIDNSDS 57
                                                                            :  * :::   .  .     *

C7_orf00015         Enterolysin A    SYSEGMFTGEYTEDLPIFEEIKGRGPITDEHARYAVGAAVKYKLLPSVILSQLGWESAYG 100
C7_orf00014         Enterolysin A    TAGGTEFNGVYTEDLPSYPEIKGVGNVPDEIAQLAVGSAVKYHLLPSVIISQWAYESEWG 78
MP1-2_orf00003      Enterolysin A    SSGGEAFTGEYSEGLPIYKEIKGRGQFSDEIAQYAVGAAVKYKLLPSVILSQYGYESAFG 117
                                     : .   *.* *:*.** : **** * . ** *: ***:****:******:** .:** :*

C7_orf00015         Enterolysin A    QSYSGKNDNNYFGITWYSGCPYPKGSARGVGGSEGGNYMKFPDAKACYSYYGYMVATQSN 160
C7_orf00014         Enterolysin A    HSASAKNDNNFFGITWFEGCPFPKGTARGVGGSEGGNYMKFPNKKSAFSYYGYMVASQTN 138
MP1-2_orf00003      Enterolysin A    TSASARNDLNYFGITWFDGCLFPKGTARGIGGIEGGWYMKFPNSKAAFSYYGFMVATQSN 177
                                      * *.:** *:*****:.** :***:***:** *** *****: *:.:****:***:*:*

C7_orf00015         Enterolysin A    FNACVGNKDPGQCLLILGRGGYAAAGITEGSAYYQGAMGIIKSYNLTEYDEFAIKKWGSI 220
C7_orf00014         Enterolysin A    FNACVGNKSPEQCLLTLGRGGYAAAGITMNSPYFTGCMSIIKSNNLTQYDDFAIKKWKDF 198
MP1-2_orf00003      Enterolysin A    FNACVGNKSPGASLLILGRGGYAAAGITEDSAYYKNCMSIINKNKLTEYDEFAIKHWGE- 236
                                     ********.*  .** ************ .* *: ..*.**:. :**:**:****:* .

C7_orf00015         Enterolysin A    GSTSSGGSTGSLGGGWYNPFLGSSLERSSFLGGQLFGKNPGGEFRVNGFHNGLDFGSVDH 280
C7_orf00014         Enterolysin A    GGN----TGGSVGGGWGWPFPDA--GQGSFAGGQLFGKNPGGEFRENGFHDGLDFGSVDH 252
MP1-2_orf00003      Enterolysin A    GGN----NNGIITGEWTNPFPGSSLDKNSFSGGQLFGTNPGGEFRPNGFHDGLDFGSVDH 292
                                     *..    . * : * *  ** .:   :.** ******.******* ****:*********

C7_orf00015         Enterolysin A    PGNEIHAVHAGTVVFAGNPSIAALGSCVIVIKDGDLSMVYQEFGSSPSNARVKVGDKVKA 340
C7_orf00014         Enterolysin A    PGNEIHAIHGGTVTYVGNPGISGLGACVIVINDSGLNMVYQEFATSTSNAKVKVGDKVKL 312
MP1-2_orf00003      Enterolysin A    PGSEIHAVHGGKVVYVGNPGISGLGACVIVINYDGLNMVYQEFANSTGNSRVKVGDQVKV 352
                                     **.****:*.*.*.:.***.*:.**:*****: ..*.******..* .*::*****:**

C7_orf00015         Enterolysin A    GQVIGIRDTAHLHLGFTKSDWYAAQASAFKNDGVWLDPLPYLQQSGGRRED 391
C7_orf00014         Enterolysin A    GDVIGIRDTEHLHLGITKKDWLQAESSAFTDDGTWLDPLKIITTGKY---- 359
MP1-2_orf00003      Enterolysin A    GQVIGIRDTAHLHLGFTRMDWRQAQGHAFIDDGTWIDPLPFLNSSKK---- 399
                                     *:******* *****:*: **  *:. ** :**.*:***  :  .
```

Figure S1: Alignment of putative **enterolysin A** (class III) sequences (first branch) from *E. hirae* genomes using Clustal Omega software. Identical residues are shaded in grey. Identity (*) 51.1 %; Strongly similar (:) 12.5 %; Weakly similar (.) 10.0 %.

GENOME	BACTERIOCIN	AA SEQUENCE	
**MP1-5**	Enterolysin A	MIESKERT**T**IMGQFETAKTIWDYLVSQGWSKTAVAGLLGNMQSESGIIADRWESDIVGNM	60
**MP1-4**	Enterolysin A	MIESKERT**I**IMGQFETAKTIWDYLVSQGWSKTAVAGLLGNMQSESGIIADRWESDIVGNM	60
**MP1-1**	Enterolysin A	MIESKERT**I**IMGQFETAKTIWDYLVSQGWSKTAVAGLLGNMQSESGIIADRWESDIVGNM	60
**C7_orf00005**	Enterolysin A	MIESKERT**T**IMGQFETAKTIWDYLVSQGWSKTAVAGLLGNMQSESGIIADRWESDIVGNM	60
**DMW1-1**	Enterolysin A	MIESKERT**T**IMGQFETAKTIWDYLVSQGWSKTAVAGLLGNMQSESGIIADRWESDIVGNM	60
**MP1-2_orf00023**	Enterolysin A	MIESKERT**T**IMGQFETAKTIWDYLVSQGWSKTAVAGLLGNMQSESGIIADRWESDIVGNM	60
		******** ***	
**MP1-5**	Enterolysin A	SGGYGLVQWTPASKYINWANANGLNYKDVISQCKRIEWEVANNQQFYNISMTFQQFKTSN	120
**MP1-4**	Enterolysin A	SGGYGLVQWTPASKYINWANANGLNYKDVISQCKRIEWEVANNQQFYNISMTFQQFKTSN	120
**MP1-1**	Enterolysin A	SGGYGLVQWTPASKYINWANANGLNYKDVISQCKRIEWEVANNQQFYNISMTFQQFKTSN	120
**C7_orf00005**	Enterolysin A	SGGYGLVQWTPASKYINWANANGLNYKDVISQCKRIEWEVANNQQFYNISMTFQQFKTSN	120
**DMW1-1**	Enterolysin A	SGGYGLVQWTPASKYINWANANGLNYKDVISQCKRIEWEVANNQQFYNISMTFQQFKTSN	120
**MP1-2_orf00023**	Enterolysin A	SGGYGLVQWTPASKYINWANANGLNYKDVISQCKRIEWEVANNQQFYNISMTFQQFKTSN	120
		**	
**MP1-5**	Enterolysin A	KSPEELANIFIQYYERPANANQPARAQQARYWYQQLNRTKVVDWFNKHRGQITYSMTGSR	180
**MP1-4**	Enterolysin A	KSPEELANIFIQYYERPANANQPARAQQARYWYQQLNRTKVVDWFNKHRGQITYSMTGSR	180
**MP1-1**	Enterolysin A	KSPEELANIFIQYYERPANANQPARAQQARYWYQQLNRTKVVDWFNKHRGQITYSMTGSR	180
**C7_orf00005**	Enterolysin A	KSPEELANIFIQYYERPANANQPARAQQARYWYQQLNRTKVVDWFNKHRGQITYSMTGSR	180
**DMW1-1**	Enterolysin A	KSPEELANIFIQYYERPANANQPARAQQARYWYQQLNRTKVVDWFNKHRGQITYSMTGSR	180
**MP1-2_orf00023**	Enterolysin A	KSPEELANIFIQYYERPANANQPARAQQARYWYQQLNRTKVVDWFNKHRGQITYSMTGSR	180
		**	
**MP1-5**	Enterolysin A	NGTDGTADCSGSITQAVKDSSGIPYSYLYNTVTLGGYLQRCGYVLVLVGNSNASNLSQLK	240
**MP1-4**	Enterolysin A	NGTDGTADCSGSITQAVKDSSGIPYSYLYNTVTLGGYLQRCGYVLVLVGNSNASNLSQLK	240
**MP1-1**	Enterolysin A	NGTDGTADCSGSITQAVKDSSGIPYSYLYNTVTLGGYLQRCGYVLVLVGNSNASNLSQLK	240
**C7_orf00005**	Enterolysin A	NGTDGTADCSGSITQAVKDSSGIPYSYLYNTVTLGGYLQRCGYVLVLVGNSNASNLSQLK	240
**DMW1-1**	Enterolysin A	NGTDGTADCSGSITQAVKDSSGIPYSYLYNTVTLGGYLQRCGYVLVLVGNSNASNLSQLK	240
**MP1-2_orf00023**	Enterolysin A	NGTDGTADCSGSITQAVKDSSGIPYSYLYNTVTLGGYLQRCGYVLVLVGNSNASNLSQLK	240
		**	
**MP1-5**	Enterolysin A	DEDIVLLSGGSSMADSGGAIGHTGVITGGGKNITSTCYYTQGEKNTAIQELRFDKNYIVA	300
**MP1-4**	Enterolysin A	DEDIVLLSGGSSMADSGGAIGHTGVITGGGKNITSTCYYTQGEKNTAIQELRFDKNYIVA	300
**MP1-1**	Enterolysin A	DEDIVLLSGGSSMADSGGAIGHTGVITGGGKNITSTCYYTQGEKNTAIQELRFDKNYIVA	300
**C7_orf00005**	Enterolysin A	DEDIVLLSGGSSMADSGGAIGHTGVITGGGKNITSTCYYTQGEKNTAIQELRFDKNYIVA	300
**DMW1-1**	Enterolysin A	DEDIVLLSGGSSMADSGGAIGHTGVITGGGKNITSTCYYTQGEKNTAIQELRFDKNYIVA	300
**MP1-2_orf00023**	Enterolysin A	DEDIVLLSGGSSMADSGGAIGHTGVITGGGKNITSTCYYTQGEKNTAIQELRFDKNYIVA	300
		**	
**MP1-5**	Enterolysin A	NGFHYYEVWRFSGGFNQSSSTG**I**TNDPTPPSFSTNVHYSLRVLGGAWLGEITNFNNSDSN	360
**MP1-4**	Enterolysin A	NGFHYYEVWRFSGGFNQSSSTG**M**TNDPTPPSFSTNVHYSLRVLGGAWLGEITNFNNSDSN	360
**MP1-1**	Enterolysin A	NGFHYYEVWRFSGGFNQSSSTG**I**TNDPTPPSFSTNVHYSLRVLGGAWLGEITNFNNSDSN	360
**C7_orf00005**	Enterolysin A	NGFHYYEVWRFSGGFNQSSSTG**I**TNDPTPPSFSTNVHYSLRVLGGAWLGEITNFNNSDSN	360
**DMW1-1**	Enterolysin A	NGFHYYEVWRFSGGFNQSSSTG**M**TNDPTPPSFSTNVHYSLRVLGGAWLGEITNFNNSDSN	360
**MP1-2_orf00023**	Enterolysin A	NGFHYYEVWRFSGGFNQSSSTG**M**TNDPTPPSFSTNVHYSLRVLGGAWLGEITNFNNSDSN	360
		**********************:*************************************	
**MP1-5**	Enterolysin A	GFSGLPNHQHDMLYIKVDKGTLRYRVHTMTSGWLDWVSKGDPNDMVNGCAGNPGEAIDGV	420
**MP1-4**	Enterolysin A	GFSGLPNHQHDMLYIKVDKGTLRYRVHTMTSGWLDWVSKGDPNDMVNGCAGNPGEAIDGV	420
**MP1-1**	Enterolysin A	GFSGLPNHQHDMLYIKVDKGTLRYRVHTMTSGWLDWVSKGDPNDMVNGCAGNPGEAIDGV	420
**C7_orf00005**	Enterolysin A	GFSGLPNHQHDMLYIKVDKGTLRYRVHTMTSGWLDWVSKGDPNDMVNGCAGNPGEAIDGV	420
**DMW1-1**	Enterolysin A	GFSGLPNHQHDMLYIKVDKGTLRYRVHTMTSGWLDWVSKGDPNDMVNGCAGNPGEAIDGV	420
**MP1-2_orf00023**	Enterolysin A	GFSGLPNHQHDMLYIKVDKGTLRYRVHTMTSGWLDWVSKGDPNDMVNGCAGNPGEAIDGV	420
		**	
**MP1-5**	Enterolysin A	QIYYTTPAGETYSQAYYRSQTTARADWLQTCCDDGTSIVGYDGWAGMFGEPLDRLQIGIA	480
**MP1-4**	Enterolysin A	QIYYTTPAGETYSQAYYRSQTTARADWLQTCCDDGTSIVGYDGWAGMFGEPLDRLQIGIA	480
**MP1-1**	Enterolysin A	QIYYTTPAGETYSQAYYRSQTTARADWLQTCCDDGTSIVGYDGWAGMFGEPLDRLQIGIA	480
**C7_orf00005**	Enterolysin A	QIYYTTPAGETYSQAYYRSQTTARADWLQTCCDDGTSIVGYDGWAGMFGEPLDRLQIGIA	480
**DMW1-1**	Enterolysin A	QIYYTTPAGETYSQAYYRSQTTARADWLQTCCDDGTSIVGYDGWAGMFGEPLDRLQIGIA	480
**MP1-2_orf00023**	Enterolysin A	QIYYTTPAGETYSQAYYRSQTTARADWLQTCCDDGTSIVGYDGWAGMFGEPLDRLQIGIA	480
		**	

**MP1-5**	Enterolysin A	KSNPLFTYSPGVNNGGSFSTNVHYGLRVLGGSWLGEITNFNDVDSNGFSGLPNNQHDMLY	540
**MP1-4**	Enterolysin A	KSNPLFTYSPGVNNGGSFSTNVHYGLRVLGGSWLGEITNFNDVDSNGFSGLPNNQHDMLY	540
**MP1-1**	Enterolysin A	KSNPLFTYSPGVNNGGSFSTNVHYGLRVLGGSWLGEITNFNDVDSNGFSGLPNNQHDMLY	540
**C7_orf00005**	Enterolysin A	KSNPLFTYSPGVNNGGSFSTNVHYGLRVLGGSWLGEITNFNDVDSNGFSGLPNNQHDMLY	540
**DMW1-1**	Enterolysin A	KSNPLFTYSPGVNNGGSFSTNVHYGLRVLGGSWLGEITNFNDVDSNGFSGLPNNQHDMLY	540
**MP1-2_orf00023**	Enterolysin A	KSNPLFTYSPGVNNGGSFSTNVHYGLRVLGGSWLGEITNFNDVDSNGFSGLPNNQHDMLY	540
		**	
**MP1-5**	Enterolysin A	IKVDAGTIRYRVHTVKSGWLDWVSKGDPNDMVNGCAGNPGEAIDGVQLYYTTPTGK**A**LSQ	600
**MP1-4**	Enterolysin A	IKVDAGTIRYRVHTVKSGWLDWVSKGDPNDMVNGCAGNPGEAIDGVQLYYTTPTGK**T**LSQ	600
**MP1-1**	Enterolysin A	IKVDAGTIRYRVHTVKSGWLDWVSKGDPNDMVNGCAGNPGEAIDGVQLYYTTPTGK**T**LSQ	600
**C7_orf00005**	Enterolysin A	IKVDAGTIRYRVHTVKSGWLDWVSKGDPNDMVNGCAGNPGEAIDGVQLYYTTPTGK**T**LSQ	600
**DMW1-1**	Enterolysin A	IKVDAGTIRYRVHTVKSGWLDWVSKGDPNDMVNGCAGNPGEAIDGVQLYYTTPTGK**T**LSQ	600
**MP1-2_orf00023**	Enterolysin A	IKVDAGTIRYRVHTVKSGWLDWVSKGDPNDMVNGCAGNPGEAIDGVQLYYTTPTGK**T**LSQ	600
		:*	
**MP1-5**	Enterolysin A	AYYRSQTTARAGWLGVCCDDGTSIAGYDGWAGMFGE**P**LDRLQI**G**IAASNQFFD	653
**MP1-4**	Enterolysin A	AYYRSQTTARAGWLGVCCDDGTSIAGYDGWAGMFGE**P**LDRLQI**G**IAASNQFFD	653
**MP1-1**	Enterolysin A	AYYRSQTTARAGWLGVCCDDGTSIAGYDGWAGMFGE**S**LDRLQI**S**IAASNQFFD	653
**C7_orf00005**	Enterolysin A	AYYRSQTTARAGWLGVCCDDGTSIAGYDGWAGMFGE**P**LDRLQI**S**IAASNQFFD	653
**DMW1-1**	Enterolysin A	AYYRSQTTARAGWLGVCCDDGTSIAGYDGWAGMFGE**P**LDRLQI**S**IAASNQFFD	653
**MP1-2_orf00023**	Enterolysin A	AYYRSQTTARAGWLGVCCDDGTSIAGYDGWAGMFGE**P**LDRLQI**S**IAASNQFFD	653
		************************************ ******.*********	

Figure S2: Alignment of putative **enterolysin A** (class III) sequences (second branch) from *E. hirae* genomes using Clustal Omega software. The differences among sequences are shaded in yellow. Identity (*) 99.2 %; Strongly similar (:) 0.3 %; Weakly similar (.) 0.2 %.

GENOME	BACTERIOCIN	AA SEQUENCE	
**Bagel: 63.3**	Enterolysin A	MKNILLSILGVLSIVVSLAFSSYSVNAASNEWSWPLGKPYAGRYEEGQQFGNTAFNRGGT	60
B9	Enterolysin A	MKNILLSILGVLSIVVSLAFSSYSVNAASNEWSWPLGKPYAGRYEEGQQFGNTAFNRGGT	60
MP5-1	Enterolysin A	MKNILLSILGVLSIVVSLAFSSYSVNAASNEWSWPLGKPYAGRYEEGQQFGNTAFNRGGT	60
MP8-1	Enterolysin A	MKNILLSILGVLSIVVSLAFSSYSVNAASNEWSWPLGKPYAGRYEEGQQFGNTAFNRGGT	60
MP9-10	Enterolysin A	MKNILLSILGVLSIVVSLAFSSYSVNAASNEWSWPLGKPYAGRYEEGQQFGNTAFNRGGT	60
RD1-1	Enterolysin A	MKNILLSILGVLSIVVSLAFSSYSVNAASNEWSWPLGKPYAGRYEEGQQFGNTAFNRGGT	60
ST1-20_orf00030	Enterolysin A	MKNILLSILGVLSIVVSLAFSSYSVNAASNEWSWPLGKPYAGRYEEGQQFGNTAFNRGGT	60
GT3-2	Enterolysin A	MKKILISVLGVLSIVVSMAFSSYSVNAASNNWSWPLGKPYAGRYEEGQQFGNTAFDRGGT	60
ST1-20_orf00016	Enterolysin A	VKKTLISVLGVLSIVVSMAFSSYSVNAASNDWSWPLGKPYAGRYEEGQQFGNTAFDRGGT	60
		:*: *:*:*********:************:************************:****	
**Bagel: 63.3**	Enterolysin A	YFHDGFDFGSAIYGNGSVYAVHDGKILYAGWDPVGGGSLGAFIVLQAGNTNVIYQEFSRN	120
B9	Enterolysin A	YFHDGFDFGSAIYGNGSVYAVHDGKILYAGWDPVGGGSLGAFIVLQAGNTNVIYQEFSRN	120
MP5-1	Enterolysin A	YFHDGFDFGSAIYGNGSVYAVHDGKILYAGWDPVGGGSLGAFIVLQAGNTNVIYQEFSRN	120
MP8-1	Enterolysin A	YFHDGFDFGSAIYGNGSVYAVHDGKILYAGWDPVGGGSLGAFIVLQAGNTNVIYQEFSRN	120
MP9-10	Enterolysin A	YFHDGFDFGSAIYGNGSVYAVHDGKILYAGWDPVGGGSLGAFIVLQAGNTNVIYQEFSRN	120
RD1-1	Enterolysin A	YFHDGFDFGSAIYGNGSVYAVHDGKILYAGWDPVGGGSLGAFIVLQAGNTNVIYQEFSRN	120
ST1-20_orf00030	Enterolysin A	YFHDGFDFGSAIYGNGSVYAVHDGKILYAGWDPVGGGSLGAFIVLQAGNTNVIYQEFSRN	120
GT3-2	Enterolysin A	YFHDGFDFGSAIYGNGSVYAVYDGKILYAGWDPVGGGSLGAFIVLQAGDTNVIYQEFSRN	120
ST1-20_orf00016	Enterolysin A	YFHDGFDFGSAIYGNGSVYAVHDGKILYAGWDPVGGGSLGAFIVLQAGDTNVIYQEFSRN	120
		*********************:**************************:***********	
**Bagel: 63.3**	Enterolysin A	VGDIKVSTGQTVKKGQLIGKFTSSHLHLGMTKKEWRSAHSSWNKDDGTWFNPIPILQGGS	180
B9	Enterolysin A	VGDIKVSTGQTVKKGQLIGKFTSSHLHLGMTKKEWRSAHSSWNKDDGTWFNPIPILQGGS	180
MP5-1	Enterolysin A	VGDIKVSTGQTVKKGQLIGKFTSSHLHLGMTKKEWRSAHSSWNKDDGTWFNPIPILQGGS	180
MP8-1	Enterolysin A	VGDIKVSTGQTVKKGQLIGKFTSSHLHLGMTKKEWRSAHSSWNKDDGTWFNPIPILQGGS	180
MP9-10	Enterolysin A	VGDIKVSTGQTVKKGQLIGKFTSSHLHLGMTKKEWRSAHSSWNKDDGTWFNPIPILQGGS	180
RD1-1	Enterolysin A	VGDIKVSTGQTVKKGQLIGKFTSSHLHLGMTKKEWRSAHSSWNKDDGTWFNPIPILQGGS	180
ST1-20_orf00030	Enterolysin A	VGDIKVSTGQTVKKGQLIGKFTSSHLHLGMTKKEWRSAHSSWNKDDGTWFNPIPILQGGS	180
GT3-2	Enterolysin A	VGDIKVSTGQTVKKGQLIGNFTSSHLHLGMTKKEWRAAHSSWNKDDGTWFNPIPILQGGS	180
ST1-20_orf00016	Enterolysin A	VGDIKVSTGQTVKKGQLIGNFTSSHLHLGMTKKEWRAAHSSWNKDDGTWFNPIPILQGGS	180
		*******************:****************:***********************	
**Bagel: 63.3**	Enterolysin A	TPTPPNPGPKNFTTNVRYGLRVLGGSWLPEVTNFNNTNDGFAGYPNRQHDMLYIKVDKGQ	240
B9	Enterolysin A	TPTPPNPGPKNFTTNVRYGLRVLGGSWLPEVTNFNNTNDGFAGYPNRQHDMLYIKVDKGQ	240
MP5-1	Enterolysin A	TPTPPNPGPKNFTTNVRYGLRVLGGSWLPEVTNFNNTNDGFAGYPNRQHDMLYIKVDKGQ	240
MP8-1	Enterolysin A	TPTPPNPGPKNFTTNVRYGLRVLGGSWLPEVTNFNNTNDGFAGYPNRQHDMLYIKVDKGQ	240
MP9-10	Enterolysin A	TPTPPNPGPKNFTTNVRYGLRVLGGSWLPEVTNFNNTNDGFAGYPNRQHDMLYIKVDKGQ	240
RD1-1	Enterolysin A	TPTPPNPGPKNFTTNVRYGLRVLGGSWLPEVTNFNNTNDGFAGYPNRQHDMLYIKVDKGQ	240
ST1-20_orf00030	Enterolysin A	TPTPPNPGPKNFTTNVRYGLRVLGGSWLPEVTNFNNTNDGFAGYPNRQHDMLYIKVDKGQ	240
GT3-2	Enterolysin A	TPTPPNPGPTNFTTNVHYGLHVLGGSWLGEVTNFNNTNDGFAGYPNRQHDMLYIKVDKGE	240
ST1-20_orf00016	Enterolysin A	TPTPPNPGPTNFTTNVHYGLHVLGGSWLGEVTNFNNTNDGFAGYPNRQHDMLYIKVDKGE	240
		*********.******:***:******* ******************************:	
**Bagel: 63.3**	Enterolysin A	MKYRVHTAQSGWLPW---	255
B9	Enterolysin A	MKYRVHTAQSGWLPWVSKGDKSDTVNGAAGMPGQAIDGVQLNYITPKGEKLSQAYYRSQT	300
MP5-1	Enterolysin A	MKYRVHTAQSGWLPWVSKGDKSDTVNGAAGMPGQAIDGVQLNYITPKGEKLSQAYYRSQT	300
MP8-1	Enterolysin A	MKYRVHTAQSGWLPWVSKGDKSDTVNGAAGMPGQAIDGVQLNYITPKGEKLSQAYYRSQT	300
MP9-10	Enterolysin A	MKYRVHTAQSGWLPWVSKGDKSDTVNGAAGMPGQAIDGVQLNYITPKGEKLSQAYYRSQT	300
RD1-1	Enterolysin A	MKYRVHTAQSGWLPWVSKGDKSDTVNGAAGMPGQAIDGVQLNYITPKGEKLSQAYYRSQT	300
ST1-20_orf00030	Enterolysin A	MKYRVHTAQSGWLPWVSKGDKSDTVNGAAGMPGQAIDGVQLNYITPKGEKLSQAYYRSQT	300
GT3-2	Enterolysin A	LKYRVHTAQSGWLAWVNKGNKNDTVNGVAGIQGQAIDGVQLNYITPKGEKLSQAYYRSQT	300
ST1-20_orf00016	Enterolysin A	LKYRVHTAQSGWLAWVNKGNKNDTVNGVAGIQGQAIDGVQLNYITSKGEKLSQAYYRSQT	300
		:************ *	
**Bagel: 63.3**	Enterolysin A	---	255
B9	Enterolysin A	TKRSGWLKVSADNGSIPGLDSYAGIFGEPLDRLQIGISQSNPF	343
MP5-1	Enterolysin A	TKRSGWLKVSADNGSIPGLDSYAGIFGEPLDRLQIGISQSNPF	343
MP8-1	Enterolysin A	TKRSGWLKVSADNGSIPGLDSYAGIFGEPLDRLQIGISQSNPF	343
MP9-10	Enterolysin A	TKRSGWLKVSADNGSIPGLDSYAGIFGEPLDRLQIGISQSNPF	343
RD1-1	Enterolysin A	TKRSGWLKVSADNGSIPGLDSYAGIFGEPLDRLQIGISQSNPF	343
ST1-20_orf00030	Enterolysin A	TKRSGWLKVSADNGSIPGLDSYAGIFGEPLDRLQIGISQSNPF	343
GT3-2	Enterolysin A	TKRSGWLKVSADNGSIPGLDSYAGIFGEPLDRLQIGISQSNPF	343
ST1-20_orf00016	Enterolysin A	TKRSGWLKVSADNGSIPGLDSYAGIFGEPLDRLQIGISQSNPF	343

Figure S3: Alignment of putative **enterolysin A** (class III) sequences (third branch) from *E. faecalis* genomes using Clustal Omega software. The differences among sequences are shaded in yellow. Identity (*) 68.8 %; Strongly similar (:) 4.4 %; Weakly similar (.) 0.3 %.

GENOME	BACTERIOCIN	AA SEQUENCE
MP1-5	Enterolysin A	--MIESKERT 8
GT3-2	Enterolysin A	-- 0
**Bagel 63.3:** ***E. faecalis***	Enterolysin A	-- 0
**Bagel 62.3:** ***E. faecalis*** **LMG2333**	Enterolysin A	-- 0
B9	Enterolysin A	-- 0
**Bagel 64.3:** ***L. acidophilus***	Enterolysin A	-- 0
C7_orf00014	Enterolysin A	MSFLLFFLGIDDSDTGGSTAGGTEFNGVYTEDLPSYPEIKGVGNVPDEIAQLAVGSAVKY 60
MP1-5	Enterolysin A	TIMGQFETAKTIWDYLVSQGWSKTAVAGLLGNMQSESGIIADRWESD---------IVGN 59
GT3-2	Enterolysin A	-- 0
**Bagel 63.3:** ***E. faecalis***	Enterolysin A	-- 0
**Bagel 62.3:** ***E. faecalis*** **LMG2333**	Enterolysin A	-- 0
B9	Enterolysin A	-- 0
**Bagel: 64.3:** ***L. acidophilus***	Enterolysin A	-- 0
C7_orf00014	Enterolysin A	HLLP--SVIISQWAYE--SEWGHSASA------KNDNNFFGITWFEGCPFPKGTARGVGG 110
MP1-5	Enterolysin A	MSGGYGLVQWT---PASKYINWANANGLNYKDVI-----SQCKRIEWEVANNQQFYNISM 111
GT3-2	Enterolysin A	---MKKILISVLG-VLSIVVSM 18
**Bagel 63.3:** ***E. faecalis***	Enterolysin A	---MKNILLSILG-VLSIVVSL 18
**Bagel 62.3:** ***E. faecalis*** **LMG2333**	Enterolysin A	---MKNILLSILG-VLSIVVSL 18
B9	Enterolysin A	---MKNILLSILG-VLSIVVSL 18
**Bagel: 64.3:** ***L. acidophilus***	Enterolysin A	-------MKFRKKI-----------------------------ITLLS----AALVSTSI 20
C7_orf00014	Enterolysin A	SEGGN-YMKFPNKKSAFSYYGYMVASQTNFNACVGNKSPEQCLLTLGR----GGYAAAGI 165
		.:
MP1-5	Enterolysin A	TFQQ-FKTSNKSPEELANIFIQYYERP----ANANQPARAQQARYWYQQLNRTKVV----162
GT3-2	Enterolysin A	AFSS-YSVNAA-------------------------------SNNWSWPLGKPYAG----42
**Bagel 63.3:** ***E. faecalis***	Enterolysin A	AFSS-YSVNAA-------------------------------SNEWSWPLGKPYAG----42
**Bagel 62.3:** ***E. faecalis*** **LMG2333**	Enterolysin A	AFSS-YSVNAA-------------------------------SNEWSWPLGKPYAG----42
B9	Enterolysin A	AFSS-YSVNAA-------------------------------SNEWSWPLGKPYAG----42
**Bagel: 64.3:** ***L. acidophilus***	Enterolysin A	T------TSASSI-VSADTITDNST---------TATETTTVKKTYKWTYPFKANDKYGV64
C7_orf00014	Enterolysin A	TMNSPYFTGCMSI-IKSNNLTQYDDFAIKKWKDFGGNTGGSVGGGWGWPFPDAGQG----220
		: .. :
MP1-5	Enterolysin A	-DWFN-KHR**G**QITYSMTGS--RNGT**DG**TA**D**CSGSITQAVKDSSGIPYSYLYNTVTLGGYL 218
GT3-2	Enterolysin A	-RYEEGQQF**G**NTAFDRGGT--Y-FH**DG**F-**D**FGS----AI-YGNGSVYAVYDGKILYAGWD 92
**Bagel 63.3:** ***E. faecalis***	Enterolysin A	-RYEEGQQF**G**NTAFNRGGT--Y-FH**DG**F-**D**FGS----AI-YGNGSVYAVHDGKILYAGWD 92
**Bagel 62.3:** ***E. faecalis*** **LMG2333**	Enterolysin A	-RYEEGQQF**G**NTAFNRGGT--Y-FH**DG**F-**D**FGS----AI-YGNGSVYAVHDGKILYAGWD 92
B9	Enterolysin A	-RYEEGQQF**G**NTAFNRGGT--Y-FH**DG**F-**D**FGS----AI-YGNGSVYAVHDGKILYAGWD 92
**Bagel: 64.3:** ***L. acidophilus***	Enterolysin A	RPMSNAQVF**G**MTNYMRSVNPPSYFH**DG**W-**D**FGH-----SEVGYSPVYAIHAGTVKKVAY- 117
C7_orf00014	Enterolysin A	-SFAGGQLF**G**KNP--GGEFRENGFH**DG**L-**D**FGS-----VDHPGNEIHAIHGGTVTYVGNP 271
		: * ** * . . :: ..: .
MP1-5	Enterolysin A	QR-----CGYVLVLVGNSNASNLSQLKDEDIVLLSG**G**SSMADSGGAIGHT**G**VIT**G**GGKNI 273
GT3-2	Enterolysin A	PVGGGSLGAFIVLQAGDTNVIYQEFSRNVGDIKVST**G**--------QTVKK**G**QLI**G**N---F 141
**Bagel 63.3:** ***E. faecalis***	Enterolysin A	PVGGGSLGAFIVLQAGNTNVIYQEFSRNVGDIKVST**G**--------QTVKK**G**QLI**G**K---F 141
**Bagel 62.3:** ***E. faecalis*** **LMG2333**	Enterolysin A	PVGGGSLGAFIVLQAGNTNVIYQEFSRNVGDIKVST**G**--------QTVKK**G**QLI**G**K---F 141
B9	Enterolysin A	PVGGGSLGAFIVLQAGNTNVIYQEFSRNVGDIKVST**G**--------QTVKK**G**QLI**G**K---F 141
**Bagel: 64.3:** ***L. acidophilus***	Enterolysin A	-GSGLGWFIWVI-SPDKYVEVYQEGFTKKSDIYVKA**G**--------QKIKA**G**QKI**G**R---- 163
C7_orf00014	Enterolysin A	GISGLGACVIVI-NDSGLNMVYQEFATSTSNAKVKV**G**--------DKVKL**G**DVI**G**I---- 318
		:: . . . . :. * : * *
MP1-5	Enterolysin A	TSTCYYTQ**G**EKNTAIQ---ELRFD-KNYIVANGFHYYEV**W**RFSGGFNQSSSTGITNDPTP 329
GT3-2	Enterolysin A	TSSH-LHL**G**M---TKK---EWRAAHSSWNKDD-----GT**W**FNPIPILQGGSTPTPPNPGP 189
**Bagel 63.3:** ***E. faecalis***	Enterolysin A	TSSH-LHL**G**M---TKK---EWRSAHSSWNKDD-----GT**W**FNPIPILQGGSTPTPPNPGP 189
**Bagel 62.3:** ***E. faecalis*** **LMG2333**	Enterolysin A	TSSH-LHL**G**M---TKK---EWRSAHSSWNKDD-----GT**W**FNPIPILQGGSTPTPPNPGP 189
B9	Enterolysin A	TSSH-LHL**G**M---TKK---EWRSAHSSWNKDD-----GT**W**FNPIPILQGGSTPTPPNPGP 189
**Bagel: 64.3:** ***L. acidophilus***	Enterolysin A	LTGSHLHL**G**LTKTDKKYINKHGFPCNNWNVDN-----GT**W**LNPISVIQKYMDKQK----- 213
C7_orf00014	Enterolysin A	RDTEHLHL**G**ITKKDWLQAESSA------FTDD-----GT**W**LDPLKIITTGKY-------- 359
		* . : .* .

MP1-5	Enterolysin A	PSFSTNVHYSLRVLGGAWLGEITNFNNSDSNGFSGLPNHQHDMLYIKVDKGTLRYRVHTM	389
GT3-2	Enterolysin A	TNFTTNVHYGLHVLGGSWLGEVTNFNNT-NDGFAGYPNRQHDMLYIKVDKGELKYRVHTA	248
**Bagel 63.3:** ***E. faecalis***	Enterolysin A	KNFTTNVRYGLRVLGGSWLPEVTNFNNT-NDGFAGYPNRQHDMLYIKVDKGQMKYRVHTA	248
**Bagel 62.3:** ***E. faecalis*** **LMG2333**	Enterolysin A	KNFTTNVRYGLRVLGGSWLPEVTNFNNT-NDGFAGYPNRQHDMLYIKVDKGQMKYRVHTA	248
B9	Enterolysin A	KNFTTNVRYGLRVLGGSWLPEVTNFNNT-NDGFAGYPNRQHDMLYIKVDKGQMKYRVHTA	248
**Bagel: 64.3:** ***L. acidophilus***	Enterolysin A	--	213
C7_orf00014	Enterolysin A	--	359

MP1-5	Enterolysin A	TSGWLDWVSKGDPNDMVNGCAGNPGEAIDGVQIYYTTPAGETYSQAYYRSQTTARADWLQ	449
GT3-2	Enterolysin A	QSGWLAWVNKGNKNDTVNGVAGIQGQAIDGVQLNYITPKGEKLSQAYYRSQTTKRSGWLK	308
**Bagel 63.3:** ***E. faecalis***	Enterolysin A	QSGWLPW---	255
**Bagel 62.3:** ***E. faecalis*** **LMG2333**	Enterolysin A	QSGWLPW---	255
B9	Enterolysin A	QSGWLPWVSKGDKSDTVNGAAGMPGQAIDGVQLNYITPKGEKLSQAYYRSQTTKRSGWLK	308
**Bagel: 64.3:** ***L. acidophilus***	Enterolysin A	--	213
C7_orf00014	Enterolysin A	--	359

MP1-5	Enterolysin A	TCCDDGTSIVGYDGWAGMFGEPLDRLQIGIAKSNPLFTYSPGVNNGGSFSTNVHYGLRVL	509
GT3-2	Enterolysin A	VSADNG-SIPGLDSYAGIFGEPLDRLQIGISQSNPF------------------------	343
**Bagel 63.3:** ***E. faecalis***	Enterolysin A	--	255
**Bagel 62.3:** ***E. faecalis*** **LMG2333**	Enterolysin A	--	255
B9	Enterolysin A	VSADNG-SIPGLDSYAGIFGEPLDRLQIGISQSNPF------------------------	343
**Bagel: 64.3:** ***L. acidophilus***	Enterolysin A	--	213
C7_orf00014	Enterolysin A	--	359

MP1-5	Enterolysin A	GGSWLGEITNFNDVDSNGFSGLPNNQHDMLYIKVDAGTIRYRVHTVKSGWLDWVSKGDPN	569
GT3-2	Enterolysin A	--	343
**Bagel 63.3:** ***E. faecalis***	Enterolysin A	--	255
**Bagel 62.3:** ***E. faecalis*** **LMG2333**	Enterolysin A	--	255
B9	Enterolysin A	--	343
**Bagel: 64.3:** ***L. acidophilus***	Enterolysin A	--	213
C7_orf00014	Enterolysin A	--	359

MP1-5	Enterolysin A	DMVNGCAGNPGEAIDGVQLYYTTPTGKALSQAYYRSQTTARAGWLGVCCDDGTSIAGYDG	629
GT3-2	Enterolysin A	--	343
**Bagel 63.3:** ***E. faecalis***	Enterolysin A	--	255
**Bagel 62.3:** ***E. faecalis*** **LMG2333**	Enterolysin A	--	255
B9	Enterolysin A	--	343
**Bagel: 64.3:** ***L. acidophilus***	Enterolysin A	--	213
C7_orf00014	Enterolysin A	--	359

MP1-5	Enterolysin A	WAGMFGEPLDRLQIGIAASNQFFD	653
GT3-2	Enterolysin A	------------------------	343
**Bagel 63.3:** ***E. faecalis***	Enterolysin A	------------------------	255
**Bagel 62.3:** ***E. faecalis*** **LMG2333**	Enterolysin A	------------------------	255
B9	Enterolysin A	------------------------	343
**Bagel: 64.3:** ***L. acidophilus***	Enterolysin A	------------------------	213
C7_orf00014	Enterolysin A	------------------------	359

Figure S4: Alignment of four different **enterolysin A** (class III) and three different references (Bagel 62.3: *E. faecalis* LMG 2333; Bagel 63.3: *E.* faecalis; and Bagel: 64.3 - *Lactobacillus acidophilus*) using Clustal Omega software. Identity (*) 1.4 %; Strongly similar (:) 1.8 %; Weakly similar (.) 2.5 %.

GENOME	BACTERIOCIN	AA SEQUENCE	
**Bagel: 186.2**	**Propionicin SM1**	MNKTHKMATLVIAAILAAGMTAPTAYADSPGNTRITASEQSVLTQILGHKPTQTEYNRYV	60
HT1-3	Propionicin SM1	------MKKFIWSMLILGSVVGSTSIVS---ADSISNSEDT-----VELSPKQ-------	39
		* .:: : :: ..:.. *: .. *: **:: : .*.*	
	**Consensus**	**------M----------------T----------I--SE-----------P-Q-------**	
**Bagel: 186.2**	**Propionicin SM1**	ETYGSVPTEADINAYIEASESEGSSSQTAAHDDSTSPGTSTEIYTQAAPARFSMFFLSGT	120
HT1-3	Propionicin SM1	---------EQVQIAVENN---DVLSDVSWALDYTQPVQIQALAKAVSDYSYSDYFSSVK	87
		::: :* . . *:.: * *.* : . .: :* :* * .	
	**Consensus**	**----------------E--------S------D-T-P---------------S-F-S--**	
**Bagel: 186.2**	**Propionicin SM1**	WITRSGVVSLSLKPRKG--------GIGNEGDERTWKTVYDKFHNAGQWTRYKNNGVDAS	172
HT1-3	Propionicin SM1	WITRDGKVSLSITPKAILTKNLPSGNGGAAHIGNAWNKLLAKHKNDKNWKN------TNG	141
		****.* ****:.*: . * .:*:.: *.:* :*.. .	
	**Consensus**	**WITR-G-VSLS-P-------------G-------W-----K-N---W-----------**	
**Bagel: 186.2**	**Propionicin SM1**	MKKQYMCHFKYG-MVKTPWNLEPHKKAADVSP---VKCN-	207
HT1-3	Propionicin SM1	MLDQYLCHAQWASGMKTPWNIEPWRPDVSYAATVAKACNP	181
		* .**:** ::. :*****:** : .. : **	
	**Consensus**	**M--QY-CH-------KTPWN-EP--------------CN-**	

Figure S5: Alignment of putative **propionicin SM1** (class III) and reference sequence using Clustal Omega software. Identical residues are shaded in grey. Identity (*) 21.3 %; Strongly similar (:) 16.9 %; Weakly similar (.) 14.0 %.

GENOME	BACTERIOCIN	SUBGROUP	AA SEQUENCE	
**Bagel: 155.2**	**Mundticin AT06**	**1**	-----------------MKKLTAKE--MSQVVGGKYYGNGVSCNKKGCSVDWGKAIGIIGNNSAANLATGGAA--GWKS---	58
MP7-18	Mundticin AT06	1	-----------------LKKLTAKE--MSQVVGGKYYGNGVSCNKKGCSVDWGKAIGIIGNNSAANLATGGAA--GWKS---	58
L8	Mundticin AT06	1	--------MECV—DMTRSKKLNLRE--MKNIVGGTYYGNGVSCNKKGCSVDWGKAISIIGNNSAANLATGGAA--GWKS---	66
**Bagel: 91.2**	**Enterocin P**	**3**	--MRKKLF-SLALIGIFGLVVTNFG--TKVDAATRSYGNGVYCNNSKCWVNWGEAKENIAG----IVISGWASGLAGMGH--	71
C7	Enterocin P	3	--------------------VTNFG--TKVDAATRSYGNGVYCNNSKCWVNWGEAKENIAG----IVISGWAYGLAGMGH--	54
DMW1-1	Enterocin P	3	--MRKKLF-SLALIGTFGLAVTNFG--TKVDAATRSYGNVVYCNNSKCWVNWEEAKENIAG----IIISGWASGLAGMGH--	71
MP1-5	Enterocin P	3	--MRKKLF-SLALIGTFGLAVTNFG--TKVDAATRSYGNVVYCNNSKCWVNWEEAKENIAG----IIISGWASGLAGMGH--	71
**Bagel: 22.2**	**Bacteriocin T8**	**4**	--MKKKVLKHCVILGILGTCLAGIGTGIKVDA-ATYYGNGLYCNKEKCWVDWNQAKGEIGK----IIVNGWVNHGPWAPRR-	74
MP1-5	Bacteriocin T8	4	MIMKKKVLKHCVILRILGTCLAGIGTGIDVDA-ATYYGNVLYCNKEKCWVNWGQSWSEGLKRWGDNLFGSFIG------GR-	74
**Bagel: 16.2**	**Bacteriocin 31**	**4**	------MKKKLVICGIIGIGFTALG--TNVEA-ATYYGNGLYCNKQKCWVDWNKASREIGK----IIVNGWVQHGPWAPR--	67
MP8-1	Bacteriocin 31	4	--MKKKLVKGLVICGMIGIGFTALG--TNVEA-ATYYGNGLYCNKQKCWVDWNKASREIGK----IIVNGWVQHGPWAPR--	71
ST1-20	Bacteriocin 31	4	--MKKKLVKALVICGMIGLGFTSLG--TNAEA-ATYYGNGLYCNKQKCSVDWNKASREIGK----IIVNGWVQHGPWAPR--	71
**Bagel: 95.2**	**Enterocin SE-K4**	**4**	--MKKKLVKGLVICGMIGIGFTALG--TNVEA-ATYYGNGVYCNKQKCWVDWSRARSEIIDRGVKAYVNGFTKVLGGIGGR-	76
GT3-2	Enterocin SE-K4	4	---------------MIGIGFTALG--TNVEA-ATYYGNGVYCNKQKCWVDWSRARSEIIDRGVKAYVNGFTKVLGGIGGR-	63
GT6-1	Enterocin SE-K4	4	--MKKKLVKGLVICGIIRIGFIALG--TNIEA-ATYYGNGVYCNSKLGTSLVRRS---------------------------	50
ST1-20_orfblast_1	Enterocin SE-K4	4	--MKKKLVKGLVICGMIGIGFTALG--MNVEA-ATYYGNGVYCNKQKCWVNWGQAWSEGVKRxxxxLFGSFS------GGRI	71
ST1-20_orf00012	Enterocin SE-K4	4	--MKKKLVKGLVICGMIGIGFTALG--MNVEA-ATYYGNGVYCNKQKCWVNWGQAWSEGVKR--------------------	57
ST1-20_orf00031	Enterocin SE-K4	4	--MKKKLVKGLVICGMIGIGFTALG--TNVEA-ATYYGNGVYCNKQKCWVDWSRARSEIIDRGVKAYVNGFTKVLGGIGGR-	76
			. . *** : **.. * * .:	
	**Consensus**		------------------------------------YGN---CN---C---W------------------------------	

Figure S6: Alignment of putative **Class IIa bacteriocins** and reference sequences using Clustal Omega software. The identity among sequences are shaded in gray (different subgroups). Cysteine residues are underlined. Identity (*) 9.2 %; Strongly similar (:) 2.6 %; Weakly similar (.) 6.6 %.

The bacteriocin subgroups is in accordance with previous proposal by Nissen-Meyer and collaborators [116] based on structure and mode-of-action of non-lanthionine-containing peptide bacteriocins produced by Gram-positive bacteria.

A)

GENOME	BACTERIOCIN	AA SEQUENCE
**Bagel: 96.2**	**Enterocin X chain alpha**	MQNVKEVSVKEMKQIIGGSNDSLWYGVGQFMGKQANCITNHPV--KHMIIPGYCLSKILG----- 58
GT3-2	Enterocin X chain alpha	MKKYKVLTEKEMKQTVGGSNDGFWERVGVGIGAGSKCYAHGGRVKGYGMIPPLCVAYGIGAAFKG 65
GT6-1	Enterocin X chain alpha	MQDYKELNEKEMKKTVGGSNDGFWERVGVGIGAGSKCYANGGSVKGYDMIPPLCVAYGVGAAFKG 65
		*:. * :. ****: :*****.:* ** :* ::* :: : :** *:: :*
	**Consensus**	M---K----KEMK---GGSND--W--VG---G----C------------IP--C-----G-----

B)

GENOME	BACTERIOCIN	AA SEQUENCE
**Bagel: 97.2**	**Enterocin X chain beta**	MKKYNELSKKELLQIQGGIAPIIV----AGLGYLVKDAWDHSDQIISGFKKGWNGGRRK 55
GT3-2	Enterocin X chain beta	-MIKKELTDKELKKINGGVFPVVPVIVGGVLTYLGKQAFEHSDQIVKGFKKGWNKY--- 55
GT6-1	Enterocin X chain beta	-MIKKELTNKELKKINGGVFPVVPVIVGGVLTYLGKQAFEHADQIGKGFKKGWNKY--- 55
		:**:.*** :*:**: *:: . * ** *:*::*:*** .*******
	**Consensus**	-----EL--KEL--I-GG--P---------L-YL-K-A--H-DQI--GFKKGWN-----

Figure S7: Alignment of putative **class IIb bacteriocins** and reference sequences using Clustal Omega software. Identical residues are shaded in grey and **GxxxG** motives are represented in red color. **(A) Alignment of enterocin X chain alpha sequences**. Identity (*) 30.8 %; Strongly similar (:) 23.1 %; Weakly similar (.) 4.6 %. **(B) Alignment of enterocin X chain beta sequences**. Identity (*) 45.5 %; Strongly similar (:) 20.0 %; Weakly similar (.) 5.5 %.

GENOME	BACTERIOCIN	AA SEQUENCE
**Bagel: 148.1**	**Carnocyclin A**	--------MLYELVAYGIAQGTAEKVVSLINAGLTVGSIISILGGVTVGLSGVFTAVKAAIAKQGIKKAIQL 64
GT6-1	Carnocyclin A	MYKQGGIFMVYDLVAYGIAQGVAEKIVGLINAGLTVGSIISIIGGATAGLAGVFAAVKAAIAKQGIKKAIQL 72
		::*********.***:*.**************:**.*.**:***:*****************
	**Consensus**	--------M-Y-LVAYGIAQG-AEK-V-LINAGLTVGSIISI-GG---GL-GVF-AVKAAIAKQGIKKAIQL

Figure S8: Alignment of putative **class II circular bacteriocin carnocyclin A** and reference sequence using Clustal Omega software. Identical residues are shaded in grey and **GxxxG** and **AxxxA** motives are represented in red color. Identity (*) 75.0 %; Strongly similar (:) 8.3 %; Weakly similar (.) 5.6 %.

GENOME	BACTERIOCIN	AA SEQUENCE
**Bagel: 76.2**	**Enterocin EJ97**	MLAKIKA**M**IK**KF**PNPY**T**LAA**KLT**T**YEI**N**W**Y**K**QQY**G**R**YPWE**R**P**VA 44
MP10-1	Enterocin EJ97	----VIS**M**KF**KF**NPTG**T**IVK**KLT**Q**YEI**A**W**F**K**NKH**G**Y**YPWE**I**P**RC 40
		`    : :*  **    *:. *** *** *:*:::* **** * .`
	**Consensus**	-------**M**--**KF**----**T**---**KLT**-**YEI**-**W**-**K**---**G**-**YPWE**-**P**--

Figure S9: Alignment of putative **class II leaderless bacteriocin enterocin EJ97** and reference sequence using Clustal Omega software. Identical residues are shaded in grey. Identity (*) 40.9 %; Strongly similar (:) 15.9 %; Weakly similar (.) 4.5 %.

A)

GENOME	BACTERIOCIN	AA SEQUENCE
**Bagel: 225.2**	**UviB**	-----MDSEL---FKLMATQGAFAILFSYLLFYVLKENSKREDKYQNIIEELTELLPK---IKEDVEDIKEKLNK 64
MP8-17	UviB	MKVGEILEEFVKGLLTNPEQISFAVLFVSLLFWVMKQNNDREQNYQKTIDKLADSLKDVESIKTTVEKINEKLN- 74
		: .*: : * :**:** ***:*:*:*..**::**: *::*:: * . ** **.*:****
	**Consensus**	**--------E----------Q--FA-LF--LLF-V-K-N--RE--YQ--I--L---L-----IK--VE-I-EKLN-**

B)

GENOME	BACTERIOCIN	AA SEQUENCE
**Bagel: 109.2**	**Enterocin 96**	---------MLNKKLLENGVVNAVTIDELDAQFGGMSKRDCNLMKACCAGQAVTYAIHSLLNRLGGDSSDPAGCNDIVRKYCK 74
GT3-2	Enterocin 96	VERTKGDNTMLNKKLLENGVVNAVTIDELDAQFGGMSKRDCNLMKACCAGQAVTYAIHSLLNRLGGDSSDPAGCNDIVRKYCK 83
DMW1-1	Enterocin 96	---------MINKKLFDSGIVNPVTIEDLDNQFGGP-KRQCSLMKACCVGQAVTYVIHNPLNQMGRDSSDPEGCNAIVRKYC- 72
		*:****::.*:** ***::** **** **:*.******.******.**. **::* ***** *** ******
	**Consensus**	**---------M-NKKL---G-VN-VTI--LD-QFGG--KR-C-LMKACC-GQAVTY-IH--LN--G-DSSDP-GCN-IVRKYC-**

C)

GENOME	BACTERIOCIN	AA SEQUENCE
**Bagel: 89.2**	**Enterocin NKR-5-3D**	MTNRKILPKEELKKIKGGTPGGIDFISGGPHVAQDVLNAIKNFFK 45
L8	Enterocin NKR-5-3D	MLKKKLVIKEEQKKIKGGTPGGFDYLTAGPHAAKGILNAIKNYF- 44
		* ::*:: *** **********:*:::.***.*:.:******:*
	**Consensus**	**M---K---KEE-KKIKGGTPG--D----GPH-A---LNAIKN-F-**

D)

GENOME	BACTERIOCIN	AA SEQUENCE
**Bagel: 209.2**	**Sakacin Q**	MQNTKELSVVELQQILGGKRA----SFGKCVVGAAGLGAGVSGGLWGMA----AGGIGGELAYMGANGCL----- 62
GT6-1	Sakacin Q	MQNVKELSSVEMQKTIGGAKWSKEQYLNTCVAGAYGAALSGAAKHWKLGPGALVGALGSEISYMSQNGCFNKNGA 75
		.* **:*: :** : :..**.** * . . :. * :. .*.:*.* ::**. ***:
	**Consensus**	**MQN-KELS-VE-Q---GG-----------CV-GA-G---------W--------G--G-E--YM--NGC------**

E)

GENOME	BACTERIOCIN	AA SEQUENCE	
**Uniprot: Q3XXB3**	**Lactococcin 972**	MKKFLCLSALMGVLLTSGGIVSATEALNLDVPEDHTAIYGGGMEATEEGFSSSKLRYAAG	60
DMW1-1	Lactococcin 972	MKKFLCLSALMGVLLTSGGIVSATEALNLDVPEDHTAIYGGGMEATEEGFSSSKLRYAAG	60
		**	
	**Consensus**	**MKKFLCLSALMGVLLTSGGIVSATEALNLDVPEDHTAIYGGGMEATEEGFSSSKLRYAAG**	
**Uniprot: Q3XXB3**	**Lactococcin 972**	GGDFNCGVNGFKVYANYYHARAKHSATAKNGRGGQVRSVQKAGVRAYATCNATLTGNTGWWNVY	124
DMW1-1	Lactococcin 972	GGDFNCGVNGFKVYANYYHARAKHSATAKNGRGGQVRSVQKAGVRAYATCNATLTGNTGWWNVY	124
		**	
	**Consensus**	**GGDFNCGVNGFKVYANYYHARAKHSATAKNGRGGQVRSVQKAGVRAYATCNATLTGNTGWWNVY**	

Figure S10: Alignment of putative **class II other bacteriocins** and reference sequences using Clustal Omega software. Identical residues are shaded in grey. **(A) Alignment of uviB sequences**. Identity (*) 37.8 %; Strongly similar (:) 21.6 %; Weakly similar (.) 6.8 %. **(B) Alignment of enterocin 96 sequences**. Identity (*) 61.4 %; Strongly similar (:) 10.8 %; Weakly similar (.) 6.0 %. **(C) Alignment of enterocin NKR-5-3D sequences**. Identity (*) 60.0 %; Strongly similar (:) 24.4 %; Weakly similar (.) 6.7 %. **(D) Alignment of sakacin Q sequences**. Identity (*) 34.7 %; Strongly similar (:) 14.7 %; Weakly similar (.) 16.0 %. **(E) Alignment of lactococcin 972 sequences.** Identity (*) 100.0 %.

References

27. Lebreton, F.; van Schaik, W.; Manson McGuire, A.; Godfrey, P.; Griggs, A.; Mazumdar, V.; Corander, J.; Cheng, L.; Saif, S.; Young, S.; et al. Emergence of epidemic multidrug-resistant *Enterococcus faecium* from animal and commensal strains. *mBio* **2013**, *4*, e00534-13, doi:10.1128/mBio.00534-13.

116. Nissen-Meyer, J.; Rogne, P.; Oppegard, C.; Haugen, H.; Kristiansen, P. Structure-function relationships of the non-lanthionine-containing peptide (class II) bacteriocins produced by Gram-Positive bacteria. CPB **2009**, 10, 19–37, doi:10.2174/138920109787048661.
